# Redox Metabolism and Vascular Calcification in Chronic Kidney Disease

**DOI:** 10.3390/biom13091419

**Published:** 2023-09-20

**Authors:** Natalia Carrillo-López, Sara Panizo, Beatriz Martín-Carro, Juan Carlos Mayo Barrallo, Pablo Román-García, Raúl García-Castro, Jesús María Fernández-Gómez, Miguel Ángel Hevia-Suárez, Julia Martín-Vírgala, Sara Fernández-Villabrille, Laura Martínez-Arias, Sara Barrio Vázquez, Laura Calleros Basilio, Manuel Naves-Díaz, Jorge Benito Cannata-Andía, Isabel Quirós-González, Cristina Alonso-Montes, José Luis Fernández-Martín

**Affiliations:** 1 Bone and Mineral Research Unit, Instituto de Investigación Sanitaria del Principado de Asturias (ISPA), Hospital Universitario Central de Asturias, 33011 Oviedo, Spain; ncarrillolopez.huca@gmail.com (N.C.-L.); sarapanizogarcia@gmail.com (S.P.); bea_m15@hotmail.com (B.M.-C.); pablo.roman.garcia@gmail.com (P.R.-G.); julia.martinvirgala90@gmail.com (J.M.-V.); sarafv0012@gmail.com (S.F.-V.); lauramartinezarias@gmail.com (L.M.-A.); sbarriovazquez@gmail.com (S.B.V.); mnaves.huca@gmail.com (M.N.-D.); cristinaam.huca@gmail.com (C.A.-M.); jlfernandez.huca@gmail.com (J.L.F.-M.); 2 Redes de Investigación Cooperativa Orientadas a Resultados en Salud (RICORS), RICORS2040 (Kidney Disease), 28029 Madrid, Spain; laura.calleros@edu.uah.es; 3 Department of Cellular Morphology and Biology, Instituto Universitario de Oncologia del Principado de Asturias (IUOPA), Instituto de Investigación Sanitaria del Principado de Asturias (ISPA), Universidad Oviedo, 33006 Oviedo, Spain; mayojuan@uniovi.es; 4 Department of Nephrology, Hospital Juaneda Miramar, Red Asistencial Juaneda, 07011 Palma de Mallorca, Spain; rgccandanedo@gmail.com; 5 UGC of Urology, Hospital Universitario Central de Asturias, Universidad de Oviedo, 33011 Oviedo, Spain; jmfernandezgomez23@gmail.com (J.M.F.-G.); hevia.urologo@gmail.com (M.Á.H.-S.); 6 Department of Surgery and Medical Surgical Specialities, Universidad de Oviedo, 33006 Oviedo, Spain; 7 Department of Systems Biology, Physiology Unit, Universidad de Alcalá, 28805 Alcalá de Henares, Spain; 8 Department of Medicine, Universidad de Oviedo, 33011 Oviedo, Spain

**Keywords:** vascular calcification, catalase, CKD, RUNX2, epigastric arteries, DIGE

## Abstract

Vascular calcification (VC) is a common complication in patients with chronic kidney disease which increases their mortality. Although oxidative stress is involved in the onset and progression of this disorder, the specific role of some of the main redox regulators, such as catalase, the main scavenger of H_2_O_2_, remains unclear. In the present study, epigastric arteries of kidney transplant recipients, a rat model of VC, and an in vitro model of VC exhibiting catalase (Cts) overexpression were analysed. Pericalcified areas of human epigastric arteries had increased levels of catalase and cytoplasmic, rather than nuclear runt-related transcription factor 2 (RUNX2). In the rat model, advanced aortic VC concurred with lower levels of the H_2_O_2_-scavenger glutathione peroxidase 3 compared to controls. In an early model of calcification using vascular smooth muscle cells (VSMCs), Cts VSMCs showed the expected increase in total levels of RUNX2. However, Cts VMSCs also exhibited a lower percentage of the nucleus stained for RUNX2 in response to calcifying media. In this early model of VC, we did not observe a dysregulation of the mitochondrial redox state; instead, an increase in the general redox state was observed in the cytoplasm. These results highlight the complex role of antioxidant enzymes as catalase by regulation of RUNX2 subcellular location delaying the onset of VC.

## 1. Introduction

Vascular calcification (VC) is one of the most common comorbidities associated with chronic kidney disease (CKD) [1]. CKD is defined as kidney damage associated with a reduction in the glomerular filtration rate for at least 3 months [2]. The loss of renal function affects serum biochemical levels of calcium, phosphorus, alkaline phosphatase, and parathormone (PTH), having a serious progressive impact on vascular, heart, bone, and parathyroid gland functions [1,3]. VC is the result of the deposition of hydroxyapatite crystals in the medial or intima layers of the arteries. It is a complex process that includes redox alterations, cell reprogramming, inflammation, and extracellular matrix degradation, among others [4]. The incidence of VC increases progressively during CKD [5], although there is some individual propensity whose causes remains unclear [3].

Redox species are one of the main mediators of cell signalling, homeostasis, and metabolism. Among reactive oxygen species (ROS), hydrogen peroxide (H_2_O_2_) is an effective secondary messenger that can diffuse to different cell compartments and the extracellular space [6]. In the context of vascular tissue, there are two redox metabolites essential for vascular homeostasis: nitric oxide, which acts mainly at the endothelium level [7], and H_2_O_2_, which regulates the proliferation, differentiation, and contractility of vascular smooth muscle cells (VSMCs) at the media layer [8]. ROS have to be contained in specific cell compartments and within a beneficial range of concentration (oxidative eustress) of antioxidant molecules (i.e., glutathione, GSH) and antioxidant enzymes, such as GSH peroxidase or catalase, which perform their functions in specific locations [6]. In pathological conditions, this balance favours ROS production, causing deleterious effects (oxidative distress).

In VSMCs, this distress causes VC. Increased levels of H_2_O_2_ alone have been shown to induce VC in VSMCs through the osteogenic transcription factor Runx2 [9]. In addition, various antioxidants have been shown to have a protective effect against calcium- and phosphorus-induced VC [10]. Catalase is the enzyme with the highest turnover rate found in nature; also, considering its affinity values, it is probably the most effective H_2_O_2_-scavenging enzyme (see the BRENDA Database). Catalase polymorphisms are associated with the prevention of arterial aging [11]. However, there are very few studies about its implications in cardiovascular disease or in CKD [12,13] and their results are inconclusive. A recent report has shown a potential protective role of catalase in VC [14]. The hypothesis of the current study posits that alterations in the levels of H_2_O_2_-scavenging enzymes, such as catalase, could play a role in the VC process. Consequently, the ensuing investigation delves into the contribution of redox metabolism to VC associated with CKD.

## 2. Materials and Methods

### 2.1. Kidney Transplant Recipient Clinical Data and Epigastric Arteria Collection

Samples of epigastric arteries were collected from 17 kidney transplant recipients (one sample from each patient). The fragments of epigastric arteries were obtained from excess tissue during the surgical kidney transplantation procedure. A section of the epigastric artery was fixed in formaldehyde 4% and embedded in paraffin for immunohistochemistry analysis. Clinical data including demographic characteristics (sex, age, smoking status, and body mass index), comorbidities (hypertension and hyperlipidaemia), treatments (time on dialysis), and biochemical parameters (serum phosphate and calcium) at the time of transplant were collected. All patients provided their informed consent to participate. The study was conducted according to the Declaration of Helsinki and approved by the Research Ethics Committee of the Principality of Asturias.

### 2.2. Rat Model: Establishment of Vascular Calcification in Wistar Rats

The rat study was performed according a previously described VC induction model [15]. Briefly, in 18 four-month-old male Wistar rats, chronic renal failure (CRF) was induced by surgical 7/8 nephrectomy, as previously detailed [16], and they were fed a high-phosphorus diet (HPD: 0.9% phosphorus, 0.6% calcium, and 20% protein content) (Panlab, Barcelona, Spain) for 20 weeks. Another 10 rats were sham-operated and fed a normal-phosphorus diet (NPD: 0.6% phosphorus, 0.6% calcium, and 20% protein content).

Rats were housed in wire cages and had ad libitum access to food and water. The protocol was approved by the Laboratory Animal Ethics Committee of the Oviedo University. Serum creatinine, urea, albumin, calcium, and phosphorus levels were measured at the end of the study.

### 2.3. Experimental In Vitro Calcification Model: Primary Culture of Mice VSMCs Overexpressing Catalase

Transgenic mice overexpressing catalase were generously provided by Dr. Arlan Richardson, Department of Cellular and Structural Biology, University of Texas Health Science Center at San Antonio. Comprehensive information regarding this catalase-overexpressing animal model has been previously published [17,18]. Primary cultures of VSMCs were obtained from the aorta of nephrectomised three-month-old male mice, C57/BLJ6 wild (WT) (N = 5) and transgenic, overexpressing the antioxidant enzyme catalase (Cts) (N = 5). Briefly, 2 to 3 mm of aorta fragments (explants) were placed into fibronectin-precoated (100 µg/mL) culture dishes with Dulbecco’s Modified Eagle’s Medium (DMEM) (Lonza, Verviers, Belgium) supplemented with 20% fetal bovine serum (Thermo Scientific HyClone, Logan, UT, USA). The five primary VSMC cultures were pooled. Primary VSMCs were grown for four passages and cryopreserved. For the experiments, the cells were thawed, and used between passages 6 and 8, with replicates performed using different cryopreserved cells. Cells were cultured in DMEM medium supplemented with fetal bovine serum at 10% to subconfluence. Subsequently, they were exposed to noncalcifying medium (non-CM), which consisted of DMEM/F12 (Lonza, Verviers, Belgium) supplemented with 0.1% albumin (BSA) or calcifying medium (CM), which included the addition of phosphate and calcium to noncalcifying medium, reaching final concentrations of 3 and 2 mM, respectively. Cells were incubated under these conditions for 4 days. All the experiments were carried out with cells below the 8th passage.

### 2.4. Analytical and Technical Procedures

#### 2.4.1. Aortic Calcification Measured in Kidney Transplant Recipient

Von Kossa staining was used for semiquantitative assessment of epigastric aortic calcifications. Tissue sections, 3 µm thick, were deparaffinised, rehydrated, and stained with an aqueous solution of 3% silver nitrate (LabKem, Dublin, Ireland) for 5 min and subsequently incubated with soda-formol solution (0.5% sodium carbonate (Merck, Darmstadt, Germany) + 25% formaldehyde 37% (Merck, Darmstadt, Germany)) for another 5 min. Following this, sections were rinsed in 5% sodium sulphate for 5 min and then exposed to a solution of 1% Ponceau S (Merck, Darmstadt, Germany) + 0.5% fuchsin acid (Merck, Darmstadt, Germany) for 20 min and then dehydrated and mounted. A modification of Becker et al.’s method [19] for semiquantitative von Kossa staining, according to calcification extent, was used. Briefly, epigastric rings were evaluated under optical microscopy using 2.5× objective. Each sample was assigned the following score: 0 = negative; 1 = microcalcifications; 2 = minicalcifications; and 3 = large calcifications + a coefficient (proportion of calcified artery wall thickness + degrees of calcified artery circumference divided by 100). Patients were grouped into negative (von Kossa score = 0) and positive (von Kossa score > 0) von Kossa staining.

Furthermore, calcium quantification in epigastric arteria aortic calcification was also measured using the o-cresolphtalein complexone method [20]. Briefly, frozen fragments of epigastric arteries were homogenised in 0.6 N HCl at 4 °C with gentle shaking for 24 h. After centrifugation, the calcium content was determined colorimetrically in the supernatants. The remaining pellet was resuspended in lysis buffer (125 mM Tris, 2% SDS, pH 6.8) for protein extraction, and quantification by DC method (Bio-Rad Laboratories, Hercules, CA, USA). Calcium content was normalised to total cell protein and expressed as µg calcium/mg protein.

Kauppila score (KS) [21] was used for semiquantitative assessment of abdominal aortic calcifications. In brief, a lateral plain lumbar X-ray that included the first through the fourth lumbar vertebrae was used to measure the severity of anterior and posterior aortic calcifications on a 0–3 scale for each lumbar segment. The results were summarised using an anteroposterior severity score that ranged from 0 to 24. X-ray images were evaluated by the same radiologist who was blinded to the patient’s data.

#### 2.4.2. Expression and Location of Catalase and RUNX2 in Epigastric Arteries

Immunohistochemistry (IHC) was performed using the EnVision FLEX Mini Kit (K8024, Agilent-Dako, Santa Clara, CA, USA) and Dako Autostainer system. Paraffin-embedded sections of epigastric arteries (3 µm thick) were deparaffinised and rehydrated. Heat epitope retrieval was performed at 95 °C for 20 min with a pH 9 (K8004, Agilent-Dako, Santa Clara, CA, USA).

Endogenous peroxidase activity was blocked using EnVision™ FLEX Peroxidase-Blocking Reagent (DM821). The sections were then incubated with polyclonal goat anticatalase antibody (SC34285, 1/100; Santa Cruz Biotechnology, Dallas, TX, USA) or polyclonal rabbit anti-RUNX2 antibody (SC10758, 1/50; Santa Cruz Biotechnology, Dallas, TX, USA) for 30 min, diluted in EnVision™ FLEX Antibody Diluent (K8006, Agilent-Dako, Santa Clara, CA, USA).

For catalase IHC, a polyclonal rabbit anti-goat (P0449) secondary antibody was used, while for RUNX2, the Secondary Dako EnVision + Dual Link System-HRP (Agilent-Dako, Santa Clara, CA, USA) was used, both for 30 min at room temperature. The signal was detected using diaminobenzidine chromogen as substrate in Dako EnVision™ FLEX/HRP (DM822, Agilent-Dako, Santa Clara, CA, USA). Sections were counterstained with haematoxylin. Negative controls were processed by omitting the primary or secondary antibody.

#### 2.4.3. Protein Analysis by 2D-DIGE

Aortas from sham-operated rats (without VC) (N = 3) and nephrectomised rats fed HPD with VC (N = 3), as determined by Von Kossa-positive staining, were pooled into 2 groups and analysed using 2-dimensional difference gel electrophoresis (2D-DIGE). 

Protein identification was carried out by LC-ESI-MS/MS on a Q-TRAP instrument (Applied Biosystems, Foster City, CA, USA) coupled with a nano-HPLC (Ultimate 3000, Dionex/LC Packings, Sunnyvale, CA, USA). Spectra were processed using MassLynx 4.0 and database searching was performed with the Mascot search engine (Matrix Science) against UniProt release 2011_11, as previously described [15].

A protein was considered identified when at least two different peptides were detected. Protein localisation and function were assigned according to PubMed and SwissProt information.

#### 2.4.4. Catalase Protein Levels and Activity in VSMCs Primary Culture

For Western blot, total proteins from WT and Cts were collected in RIPA buffer and quantified by Bradford assay (Bio-Rad). Equal protein amounts (30 µg) were subjected to electrophoresis and transferred to PVDF using SDS-PAGE according to standard procedures. After a 1 h incubation in a 3% BSA-TRIS blocking solution, protein detection was performed using specific antibodies for catalase (sc-34285) and beta-actin (MS-1295-P, Neomarkers, Portsmouth, NH, USA). 

Membranes were incubated overnight at 4 °C at 1:5000 dilution and 1:8000 dilution, respectively. Secondary antibodies were incubated for 1 h at room temperature (1:10,000 dilution rabbit anti-goat IgG for catalase, Cat: 401504, Calbiochem; 1:10,000 dilution goat anti-mouse IgG for beta-actin, Cat: 401215, Calbiochem). Chemiluminescence signals were digitised and quantified with a ChemiDoc™ XRS+ instrument (Bio-Rad, Hercules, CA, USA) and the ImageLab™ software v.3.0 (Bio-Rad Laboratories, Hercules, CA, USA). 

Densitometric values of catalase are presented as a ratio of the corresponding actin values on the same line.

The basal activity of catalase was measured in VSMC from WT and Cts using the commercial kit “Catalase Assay Kit” (Cayman Chemical, Ann Arbor, MI, USA), following the manufacturer’s established protocol.

#### 2.4.5. RUNX2 in VSMCs Primary Culture

Mice cells, both WT and Cts, cultured with non-CM and CM (as detailed in Section 2.3), were fixed with methanol: ethanol (1:1) for 10 min and treated with 0.1% Tween 20 + 5% BSA for 1 h at room temperature. Then, the cells were incubated overnight at 4 °C with a rabbit anti-RUNX2 polyclonal antibody (1:50 dilution; sc-10758, Santa Cruz Biotechnology, Dallas, TX, USA), followed by anti-rabbit Alexa Fluor 594 (1:400 dilution; A21207, Cell Signaling Technology, Danvers, MA, USA). The cells were counterstained with 4′,6-diamidino-2-phenylindole (DAPI) added to the mounting medium (SlowFade™ Diamond Antifade Mountant with DAPI, ThermoFisher, Waltham, MA, USA).

#### 2.4.6. Quantification of Calcium Content in VSMCs Primary Culture

VSMCs cultured with non-CM and CM were washed with PBS and then homogenised in 0.6 N HCl. Calcium content was determined by the o-cresolphtalein complexone method, as described in Section 2.4.1.

#### 2.4.7. Redox Metabolism Probes in VSMCs Primary Culture

Redox state was assessed in the cytoplasm and in the mitochondria using Dichlorofluorescein-di-acetate (DCF, Invitrogen, Waltham, MA, USA) and Di-hydro-rhodamine 123 (DHR123, Invitrogen, Waltham, MA, USA), respectively.

VSMC incubated with calcium and phosphorus or under control conditions (as described in Section 2.3) were washed with Hank’s solution and then incubated for 30 min (darkness, 36 °C, and 5% CO_2_) in a Hank’s solution containing both 500 nM DCF and 10 μM DHR123. Five confluent fields of each experimental replicate were acquired, first for DCF and then for DHR123, alternating non-CM and CM conditions to minimise bias.

### 2.5. Imaging Analyses

Images of epigastric arteries were acquired using a light microscope (DMRXA2, Leica Microsystems, Wetzlar, Germany). In VSMCs analysis, fluorescent images were captured using a Nikon Eclipse TS100 microscope.

#### 2.5.1. Catalase in Epigastric Arteries

Whole slides were scanned in a Hammamatsu NanoZoomer (20× magnification) and image analysis was performed using QPath (v 0.3.2). Images were defined as Brightfield-HDAB.

For the analysis, noncalcification and pericalcification ROIs in the media layer were manually selected using the wand tool. A pixel classifier was then applied to ROIs (threshold DAB channel 0.14 at low (7.06 μm/px) resolution). Percentage of catalase positive staining area was obtained.

#### 2.5.2. RUNX2 in Epigastric Arteries

The ratio between positive nuclei for RUNX2 and the total number of nuclei was calculated in each whole epigastric artery section (20× magnification). 

For the quantification of total protein levels, FIJI software v.153t (NIH, Bethesda, MD, USA) was used. Images were deconvoluted using the H-DAB vector. A threshold of 171 was applied to the image corresponding to the DAB channel. An ROI was draw on the original image to encompass the media layer surface and applied to the DAB channel to measure the positive RUNX2 area.

In the images where calcification was observed, the pericalcification area was defined as the proportion of media layer surrounding the length of the calcification. Noncalcification area was quantified in the same image when a safe margin of 50 microns was between pericalcification and the noncalcified area. Positive area quantification was performed as described for total protein levels. 

For nuclear quantification, the hematoxilin channel from the H-DAB deconvolution (which marked nuclei) was used as an ROI mask in the DAB channel, obtaining the positive DAB areas in nuclear ROI only.

#### 2.5.3. RUNX2 in VSMCs Primary Culture

Micrographs were analysed using FIJI software v1.53t (NIH, Bethesda, MD, USA) [22]. For total Runx2 levels, all the images were adjusted for brightness and contrast at 36 and 168, respectively. 

For total fluorescence intensity determination, the entire cell contour was outlined as a single ROI, and average fluorescence intensity was measured. Quantification of nuclear RUNX2 staining area was generated using a nuclear ROI derived from the DAPI staining (blue channel). The RUNX2 images were converted to black and white, and a threshold of 16 was applied to the images. Positive pixel area of RUNX2 was quantified within the DAPI-based ROI only.

#### 2.5.4. Redox Metabolism Probes in VSMCs Primary Culture

Micrographs were captured when cells reached full confluency, and the analysis was performed using FIJI software v1.53t. The protocol closely followed the one described in Section 2.5.3.

Total DCF and DHR123 levels were measured without applying, intensity correction. Mean fluorescence per area was measured. For the quantification of positive areas, cells were converted to BW and a threshold of 0.23 was applied. The ROI encompassed the entire field, allowing for the quantification of the total field.

### 2.6. Statistical Analysis

For statistical analysis and graphs generation, R software v 4.2.2 (R foundation for statistical computing, Vienna, Austria) and Prism GraphPad v 9.2.0 (Dotmatics, Boston, MA, USA) were used. 

The sample size was calculated considering an anticipated strong influence of calcification. With an effect size of 2, a statistical power of 80%, and a significance level (α-value) of 0.05, the necessary sample size was determined to be 6 per group. To account for possible tissue-processing losses in the case of epigastric arteries, a total of 17 individuals were included in the study. 

Regarding the number of animals used in the study of protein expression using 2D-DIGE, three rats per group were selected to manage the cost of the analysis. Consequently, we selected three rats with a high degree of calcification, as confirmed by von Kossa staining, along with another three rats as control. 

All experimental groups were tested for normality by using Shapiro–Wilk and Kolmogorov–Smirnov tests. When either or both of these tests were positive for all experimental groups, the samples were considered to follow a normal distribution and analysed by *t*-test (paired or unpaired in each case). In cases where one or more experimental groups did not follow a normal distribution, Kolmogorov–Smirnov for cumulative distributions or Wilcoxon matched pairs test were used. All values are shown as mean ± standard deviation (SD) unless otherwise indicated.

## 3. Results

### 3.1. Catalase and RUNX2 Protein Levels in Epigastric Arteries of Kidney Transplant Recipients

This study included a total of 17 epigastric arteries obtained from kidney transplant recipients. The main characteristics of the patients are summarised in Table 1. Epigastric arteries were classified as either calcified or noncalcified based on von Kossa staining (Supplementary Figure S1A).

There were no significant differences in age, sex, smoke habit, body mass index (BMI), arterial hypertension, hyperlipidaemia, time on dialysis, serum phosphate, or serum calcium levels among groups. Both the Kauppila score and epigastric calcium content showed a significant association with von Kossa grouping, providing further support for the chosen methodology in the staging process.

As we previously mentioned, earlier in vitro studies found a protective effect of catalase in vascular calcification induced by calcium and phosphorous [14]. Low levels of catalase would help to explain why certain CKD patients develop vascular calcification. To validate the relevance of these findings, the levels of catalase protein in the media layer of epigastric arteries from kidney transplant recipients were analysed using IHC. The results did not show a relationship between catalase levels in the arteries and the calcium content determined by von Kossa staining or Kauppila score in patients (Table 1).

**Figure 1:**
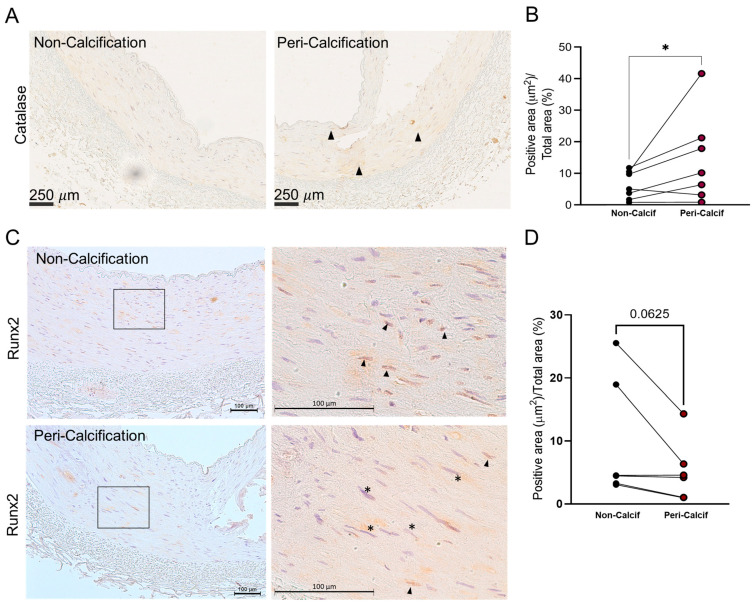
Catalase and RUNX2 protein levels in epigastric arteries from kidney transplant recipients which tested positive for von Kossa staining (calcified arteries). Noncalcified and pericalcified areas within the same artery were subjected to comparison, thus using each artery as its own control. (**A**) Catalase immunostaining (arrow heads shows localised staining). (**B**) Catalase-positive immunostaining quantification (N = 7). (**C**) RUNX2 immunostaining. Right: zoomed areas indicated on left images (square) showing nuclear Runx2 staining, positive black arrow heads, negative (*). (**D**) RUNX2-positive immunostaining quantification (N = 6). * = *p*-value < 0.05; Wilcoxon paired test. Peri-Calcif. means pericalcified area; Non-Calcif. means noncalcification area.

When possible, the levels of catalase surrounding the calcification were also analysed. Interestingly, when we compared the average levels of catalase in the media layer of the area surrounding the calcified tissue, catalase was significantly increased within pericalcified areas (*p*-value = 0.046) (Figure 1A,B). This increase was localised to specific areas; therefore, it might be circumscribed to individual cells (indicated by arrow heads). This result suggests a dual expression pattern of catalase within the same tissue, depending on the degree of calcification in the area.

In addition, RUNX2 levels were also analysed in these 17 epigastric arteries (Table 1). There was no significant increase in RUNX2 in either the calcified or noncalcified groups, either in total or nuclear levels. When the protein levels of RUNX2 were studied in pericalcification areas, no changes in total protein were observed. However, nuclear RUNX2 staining showed a decreasing trend in the pericalcification areas (*p*-value = 0.062) (Figure 1C,D and Supplementary Figure S1B). The results showed that, in pericalcification areas, where catalase levels were higher, RUNX2 levels did not change but there was a tendency toward cytoplasmic, rather than nuclear, staining.

### 3.2. Differential Protein Expression in Aortas from Rats with and without VC

To characterise general features of VC associated with CKD, we conducted a study using a rat animal model of VC induced by CRF and an HPD (see Section 2).

Rats with VC showed impairment of renal function and mineral metabolism, as measured by higher levels of serum creatinine, urea, P, lower serum albumin, and no changes in serum calcium compared to rats without VC (No-VC) (Supplementary Table S1).

Proteomic analysis was performed in aortas. 2D-DIGE analysis detected approximately 3400 protein spots, of which 77 were differentially expressed in calcified and noncalcified aortas. At least two peptides were identified in 26 of the 77 proteins differentially expressed, and five of them were directly or indirectly related to oxidative stress (Table 2). Of the five, three were directly associated with mitochondrial redox metabolism: superoxide dismutase (SODM), GSH peroxidase 3 (GPX3), and glutathione-S-transferase type Mu2 (GSTM2). In calcified aortas, there was an increase (2.14-fold change) in mitochondrial superoxide dismutase (SODM) compared to noncalcified ones. Another significant difference between calcified and noncalcified samples was observed in the metabolism of the main antioxidant glutathione. There was a significant decrease in GPX3 levels in calcified aortas, which has an analogous role to catalase in scavenging extracellular H2O2. GSTM2, which is also involved in xenobiotic metabolism and detoxification, showed a decrease in calcified tissue.

**Table 2:** Differential protein expression pattern in calcified compared with noncalcified aortas, shown as average ratio. Protein spots differentially expressed shown in Figure 2 were identified by LC-MS/MS (Supplementary Table S2 and supplementary annexe) (N = 6; N = 3 with VC and N = 3 without VC).

Spot N°	MASCOT-Symbol	Name	Accession N°	Function	Localisation	*t*-Test *p*-Value	Average Ratio	Score	MW (Da)	pI	Number of Peptides Identified
1	CAH3	Carbonic anhydrase 3	P14141	Carbonate dehydratase activity.	Cytoplasm	0.0042	4.86	95	29,413	6.89	3
2	GPX3	Glutathione peroxidase 3	P23764	Protects cells and enzymes from oxidative damage, by catalysing the reduction in hydrogen peroxide, lipid peroxides, and organic hydroperoxide, by glutathione.	Extracellular space	0.0052	−2.76	97	25,637	8.26	2
3	GSTM2	Glutathione S-transferase Mu2	P08010	Conjugation of reduced glutathione to a wide number of exogenous and endogenous hydrophobic electrophiles. Participates in the formation of novel hepoxilin regioisomers.	Cytoplasm	0.0018	−2.38	234	25,857	6.9	7
4	SODM	Superoxide dismutase [Mn], mitochondrial precursor	P07895	Destroys superoxide anion radicals which are normally produced within the cells and which are toxic to biological systems.	Mitochondrion matrix	0.0087	2.14	113	24,887	8.96	2
5	TKT	Transketolase	P50137	Catalyses the transfer of a two-carbon ketol group from a ketose donor to an aldose acceptor, via a covalent intermediate with the cofactor thiamine pyrophosphate.	Intracellular membrane	0.0041	−1.62	43	68,342	7.23	2

### 3.3. In Vitro Model of Vascular Calcification in VSCMs Overexpressing Catalase

Primary cell cultures of VSMCs were obtained from WT and transgenic overexpressing Cts mice. VMSC exhibited a 12-fold increase in Cts protein levels and a concomitant increase in Cts activity (Supplementary Figure S2A,B); similar results were reported elsewhere [14].

**Figure 3:**
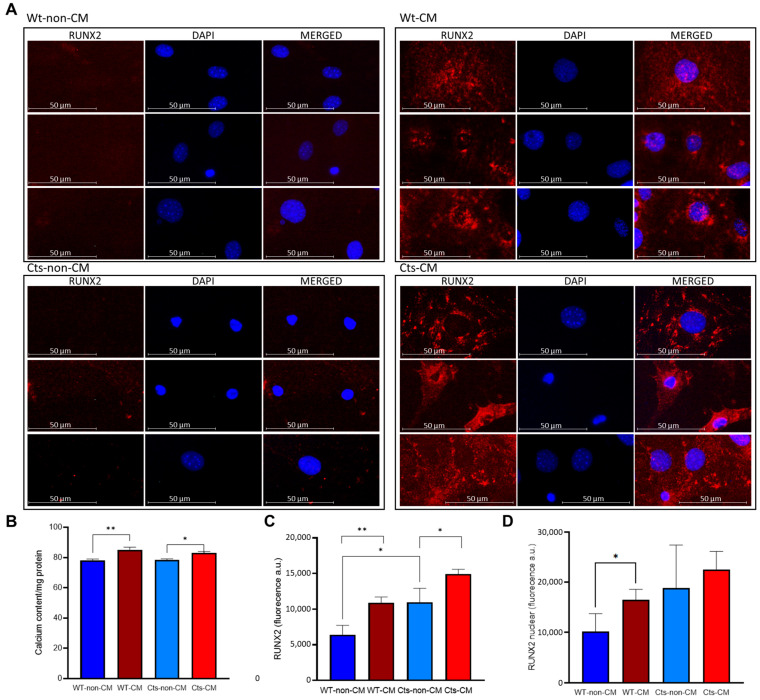
RUNX2 and calcium content in primary cultures of VSMCs from WT and catalase (Cts) overexpressing mice exposed to non-CM and CM. (**A**) Immunocytochemistry of Runx2 (red) in WT and Cts VSMC exposed to non-CM and CM; DAPI (blue) is used as nuclear counterstaining. (**B**) Calcium content in non-CM and CM of WT and Cts VSMCs. (**C**) Total Runx2 fluorescence quantification. (**D**) Nuclear Runx2 fluorescence quantification. All panels show mean ± SD. (**B**,**D**) * = *p*-value < 0.05; ** = *p*-value < 0.01. (**B**–**D**) Unpaired *t*-test. The primary cultures of VSMCs were obtained from a pool consisting of 5 wild-type mice and 5 mice overexpressing catalase. Each experiment was carried out in triplicate.

When incubated with CM, both WT and Cts VSMCs showed a significant increase in calcium content, although the differences were less pronounced in the Cts group (Figure 3B).

Cts VSMCs showed significantly higher levels of RUNX2 compared with WT cells under non-CM conditions. Furthermore, RUNX2 expression was also significantly increased in both WT and Cts after exposure to CM (Figure 3A,C). However, the percentage of the nucleus stained for RUNX2 was significantly higher in WT cells cultured with CM compared to WT cells cultured with non-CM (Figure 3A,D). This increase was not observed in Cts-CM. Negative controls for rabbit anti-RUNX2 primary and anti-rabbit Alexa Fluor 594 secondary antibody are provided (Supplementary Figure S3).

**Figure 4:**
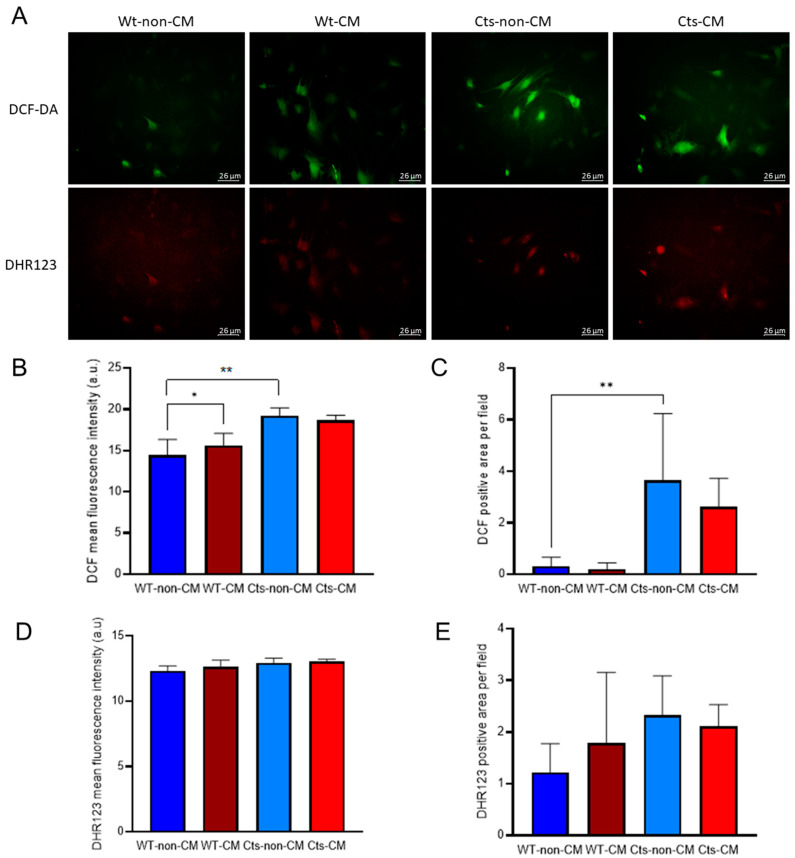
Redox state in VSMC primary cultures of VSMCs from WT and catalase (Cts) overexpressing mice exposed to non-CM and CM. (**A**) Micrographs of cytoplasmic redox state monitored with DCF-DA (green) and DHR123 (red) in VSMC cultured with non-CM and CM. (**B**) DCF-DA average fluorescence intensity. (**C**) DCF-DA positive area (px). (**D**) DHR123 average fluorescence intensity. (**E**) DCF-DA positive area(px). All panels show mean ± SD. (**B**,**C**) * = *p*-value < 0.05; ** = *p*-value < 0.01; Kolmogorov–Smirnov test. The primary cultures of VSMCs were obtained from a pool consisting of 5 wild-type mice and 5 mice overexpressing catalase. Each experiment was carried out in triplicate.

The basal cytoplasmic redox state, as measured by the average fluorescence intensity and positive area of DFC, was higher in Cts cells compared with WT cells (Figure 4A–C). Fluorescence intensity was greater in WT cells cultured with CM compared with non-CM, although there were no differences in the fluorescence area. Noteworthy, the increase found in the fluorescence intensity is based in a subset of cells which drastically increased their fluorescence, while the majority of the culture did not show changes in redox state (Figure 4A). In contrast, Cts cells showed no differences between CM and non-CM in both fluorescence intensity and fluorescent area (Figure 4B,C).

No significant changes were observed in mitochondrial redox state, measured by DHR123 fluorescence intensity, in either WT or Cts VSMCs under non-CM or CM conditions (Figure 4A,D,E).

## 4. Discussion

The initial perception of VC as a passive deposit of hydroxyapatite crystals was replaced two decades ago by a more intricate framework, unveiling a much more complex scenario. Indeed, the oxidative stress theory within the context of VC was formerly perceived as either wholly positive or entirely negative. The rise in free radicals has been associated with VC, while the absence or low levels of these radicals has been associated with a reduced incidence of VC. Nevertheless, just as observed with the former theory, the latter might also represent an oversimplification of the role of oxidative stress and redox metabolism.

In the present study, calcified epigastric arteries from CKD patients undergoing kidney transplant showed a moderate and localised increase in catalase, a crucial scavenger for H_2_O_2_, within the regions encompassing the calcification. Furthermore, there was a nonsignificant downward trend in RUNX2. These pericalcification regions likely showed a heterogeneous territory, where cells may be attempting to shield themselves from the calcification process. These areas showed a lower degree of calcification, primarily because the main areas of VC were lost during the tissue processing.

In an animal model of VC, differential expression of various proteins between calcified and noncalcified aortas revealed deregulation of several proteins associated with oxidative stress. Catalase was not detected, but a reduction in a similar enzyme, GPX3, was observed in calcified aortas. The decreased levels of extracellular H_2_O_2_ scavengers found in the aortas would be associated with the severity of VC found. It is important to emphasise that these results refer to protein levels; consequently, further confirmation of these changes at enzymatic activity level would be ideal. Nevertheless, due the limited sample quantity, especially in the human studies, conducting further analyses was not feasible.

To gain a better understanding of the role of catalase, an in vitro model was used. An increase in calcium deposits and RUNX2, both at the total protein and nuclear levels, was observed in WT cells exposed to CM. Nonetheless, when catalase was overexpressed and cultured with CM, a slight increase in calcium content was found, but it was not accompanied by a significant concurrent rise in nuclear RUNX2. The decrease in nuclear RUNX2 could partially prevent the onset of VC. This finding aligns with the results observed in epigastric arteries from CKD patients. A significant finding was the fact that calcium content and total protein levels of RUNX2 increased when catalase was overexpressed in VSMC cultured under non-CM conditions, suggesting that catalase might have a direct impact on calcium metabolism (Supplementary Figure S2C).

This might not be a mere coincidence, as H_2_O_2_ has the capability to influence calcium release from the primary intracellular calcium storage sites, namely, mitochondria and endoplasmic reticulum. It can enhance calcium release from both storage organelles and facilitate calcium uptake from the extracellular space, ultimately leading to an increase in cytoplasmic calcium content [23]. Hence, the regulation of antioxidant enzymes specialised in the elimination of H_2_O_2_, such as catalase or GPX3, as previously described, could potentially enable the maintenance of comparable levels of intracellular calcium while keeping it confined within the calcium storage organelles. In the case of GPX3, particularly its extracellular form, it may also have the capacity to inhibit the calcium uptake from the extracellular space. Maintaining a precise and controlled concentration range of catalase or peroxidase could empower cells to regulate intracellular calcium levels, ultimately preserving cell stiffness and favouring a contractile phenotype [24]. The current findings suggest that catalase might have the potential to mitigate VC even in the presence of elevated calcium levels and increased RUNX2 expression, particularly during the early stages of the process.

This counterbalance effect is not permanent, and with prolonged exposure to a calcification stimulus, the pericalcification areas observed in the epigastric arteries may undergo complete calcification. Indeed, the rat model of severe VC showed the absence of antioxidant effects, and there was a decrease in the expression of GPX3 and GST. Furthermore, consistent with this observation, other authors have also found that oxidative stress can induce VSMCs to differentiate into osteoblast [25,26].

Although no significant correlation was found between total catalase levels and von Kossa staining (*p*-value: 0.097) or with KS (*p*-value: 0.6778), our study reveal a localised increase in catalase in the vicinity of the calcification lesion in specific cells. This increase was unexpected, as previous reports described elevated catalase levels within the calcification lesions [27]. To interpret our results, two arguments can be considered. The first is that the previously mentioned study was made on a rabbit model of aortic valve calcification that did not involve renal damage. In this model, even though there was evidence of free radical production, it seems that the oxidative stimuli originated from mitochondria. Conversely, in VC associated with CKD, the stimuli seem to have a robust cytoplasmic origin, likely caused by the NOX enzymatic system [4,28]. The second argument is based on our in vitro model, which showed that the overexpression of catalase, a peroxisome-confined enzyme, does not necessarily lead to a reduction in cytoplasmic redox state.

In the presence of superoxide radical production by the NOX enzymatic system, catalase would require assistance from the CuZnSOD to convert superoxide on H_2_O_2_, a neutral molecule capable of passing through the peroxisome membrane to reach the catalase. 

This could hold particular significance in the case of VSMCs, where NOX enzymes are constitutively expressed, as they play a role in vesicle trafficking and muscle contraction [29]. The excess of superoxide might favour its reaction with other radical groups, resulting in the formation of compounds like NO or Fe^3+^–H_2_O, forming peroxynitrite or hydroxyl radicals which will also produce DCF-DA fluorescence.

A previous study showed that catalase overexpression prevented both calcium deposition and the increase in RUNX2 levels in VSMCs cultured with CM for 8 days [14]. Nevertheless, the precise mechanisms through which catalase exerted this preventive effect on calcification were not thoroughly elucidated. The current study provided us with the opportunity to study the calcification process at earlier stages, specifically 4 days of culture, before the actual onset of calcification. During the early stage of the calcification process, characterised by only an 8% increase in calcium content, no differences were observed in mitochondrial oxidative stress levels when compared to the WT group. The total levels of RUNX2 increased in both WT and Cts cells cultured with CM. Additionally, as previously mentioned, RUNX2 levels were also increased in Cts cells, even in the absence of CM. Nevertheless, it is worth noting that nuclear RUNX2 did not increase significantly in the Cts cells when cultured with CM.

These findings suggest that catalase overexpression may block the translocation of RUNX2 to the nucleus, thereby hindering the transdifferentiation process from VSMCS into osteoblast-like cells. Our working hypothesis is that during these early stages, both cell lines respond to CM stimuli, but catalase serves to delay VC by inhibiting the nuclear translocation of RUNX2. Nonetheless, it is highly improbable that such levels can be observed in nonmanipulated tissues. Therefore, if the calcification stimuli persist, the antioxidant response, including enzymes like catalase and peroxidase, would likely become overwhelmed, leading to the occurrence of calcification.

Hence, the intricacies of the relationship between catalase and calcium within the context of the muscular media layer create a very complex scenario that extends beyond the role of a simple antioxidant scavenging enzyme. This complexity may offer insight into understanding the results obtained in previous studies that explored the potential protective role of catalase in CKD and in vascular diseases [12,13], where no positive results were obtained, despite catalase being the most efficient scavenger of H_2_O_2_ in the vasculature. Both CKD and VC exhibit a very strong oxidative stress component.

Ideally, the findings presented in this study should be validated using a larger cohort of patients. For this current study, a bigger epigastric aortic tissue sample for the integration of various techniques would have constituted a valuable contribution. This would have allowed us to overcome some technical limitations of the study regarding the subcellular localisation of RUNX2 when using conventional fluorescence microscopy, which solely analyses a two-dimensional plane. Confocal microscopy would offer improved capabilities for assessing the subcellular localisation of RUNX2. Furthermore, the levels of catalase protein were measured, but enzymatic activity or other markers for redox modification such as peroxidation could not be assessed.

## 5. Conclusions

This study reveals the presence of an antioxidant response to vascular calcification within the vasculature, specifically in vascular smooth muscle cells (VSMCs), achieved by increasing H_2_O_2_-scavenging enzymes. This response appears to be localised and temporary, potentially retarding vascular calcification by influencing the subcellular localisation of RUNX2. Further studies are required to elucidate the mechanisms by which catalase could hinder the translocation of RUNX2 to the nucleus, as well as to understand the role of interactions involving enzymes beyond catalase that are also implicated in the oxidative stress scenario.

## Figures and Tables

**Figure 2:**
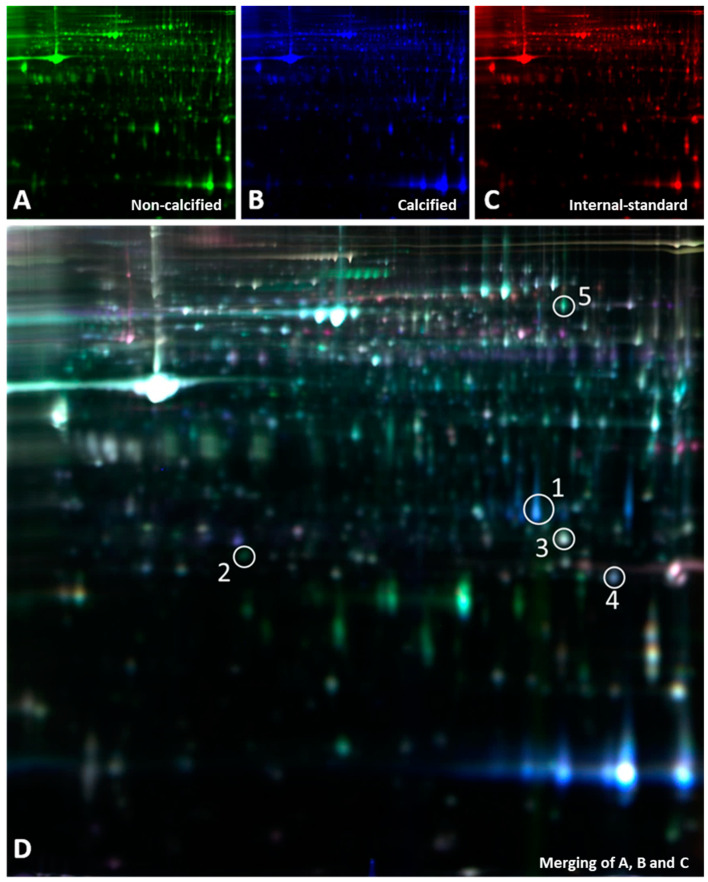
2D-DIGE images showing differentially expressed proteins between calcified and noncalcified aortas in the rat experimental model. (**A**) Cy5-labeled proteins from noncalcified aortas; (**B**) Cy2-labelled proteins from calcified aortas; (**C**) Cy3 dye staining of the internal standard; and (**D**) the merging of (**A**–**C**). The circled spots represent proteins that were differentially expressed and were directly or indirectly related to oxidative stress, as listed in Table 2. (N = 6; N = 3 with VC and N = 3 without VC).

**Table 1:** Patient characteristics classified according to von Kossa staining.

	Von Kossa Staining		
	Negative	Positive	*p*-Value
n	7	10	
Sex = Male (%)	7 (100.0)	9 (90.0)	1.000
Age (years) (mean (SD))	60.6 (7.9)	57.5 (7.0)	0.412
Smoking habit = Yes (%)	1 (14.3)	2 (20.0)	1.000
BMI (kg/m^2^) (mean (SD))	26.8 (5.0)	25.4 (5.4)	0.588
Hypertension = Yes (%)	7 (100.0)	9 (90.0)	1.000
Hyperlipidaemia = Yes (%)	4 (57.1)	5 (50.0)	1.000
Time on dialysis (months) (mean (SD))	17.1 (12.5)	30.3 (14.2)	0.068
Serum phosphate (mg/dL) (mean (SD))	4.2 (1.2)	4.2 (1.1)	0.999
Serum calcium (mg/dL) (mean (SD))	9.1 (0.8)	8.6 (0.5)	0.147
Kauppila score (mean (SD))	2.3 (2.8)	9.0 (6.0)	0.015
Von Kossa score (mean (SD))	0.0 (0.0)	3.9 (2.4)	0.001
Calcium content (ug/mg protein) (mean (SD))	15.3 (2.1)	957.2 (993.1)	0.038
Catalase protein levels (A.U.) (mean (SD))	11.2 (6.6)	9.5 (11.3)	0.738
RUNX2 protein levels (A.U.) (mean (SD))	0.9 (0.1)	0.7 (0.2)	0.076
RUNX2 positive nuclei/Total nuclei (mean (SD))	0.1 (0.1)	0.2 (0.2)	0.681

BMI: body mass index; A.U.: arbitrary units.

## Data Availability

The data presented in this study are available on request from the corresponding authors.

## Supplementary Materials

The following supporting information can be downloaded at: https://www.mdpi.com/article/10.3390/biom13091419/s1, Figure S1: Representative images of von Kossa staining. (A) Epigastric arteria negative and positive for von Kossa staining. (B) Micrographs of distant and pericalcification areas of the same epigastric arteria section for von Kossa staining. Figure S2: VSMCs from wild-type (WT) and catalase (Cts) cell culture. (A) Relative optical density of western-blot data for catalase protein expression. (B) Cts activity in VSMC cell culture. (C) Calcium content per mg of protein in WT cell culture and Cts overexpressing cells. * = *p*-value < 0.05; *** = *p*-value < 0.001. (pools of primary VSMCs were obtained from N = 5 wt and N = 5 overexpressing catalase mice, 2 independent experiments with 3 replicates were analysed). Figure S3: Immunofluorescence of RUNX2 in VSMCs. Negative controls for primary (rabbit anti-RUNX2) and secondary (anti-rabbit Alexa Fluor 594) antibody. DAPI was used as nuclear counterstaining. Table S1: Biochemical parameters of sham-operated rats with normal renal function without vascular calcification (No-VC) and nephrectomised rats fed a high-phosphorus diet (HPD) with vascular calcification (VC) at 20 weeks. Table S2: Summary of the peptides identified by LC-MS/MS. More detailed information can be found in the annexe.
