# Supplementary material for: Redox Metabolism and Vascular Calcification in Chronic Kidney Disease
Source: Biomolecules. 2023 Sep 20;13(9):1419. doi: 10.3390/biom13091419 (PMC10526886; doi:10.3390/biom13091419)
Supplement: Supplementary file 1 [file biomolecules-13-01419-s001.zip › biomolecules-2544850-supplementary.pdf]

Supplementary material

Redox metabolism and vascular calcification in Chronic-Kidney Disease

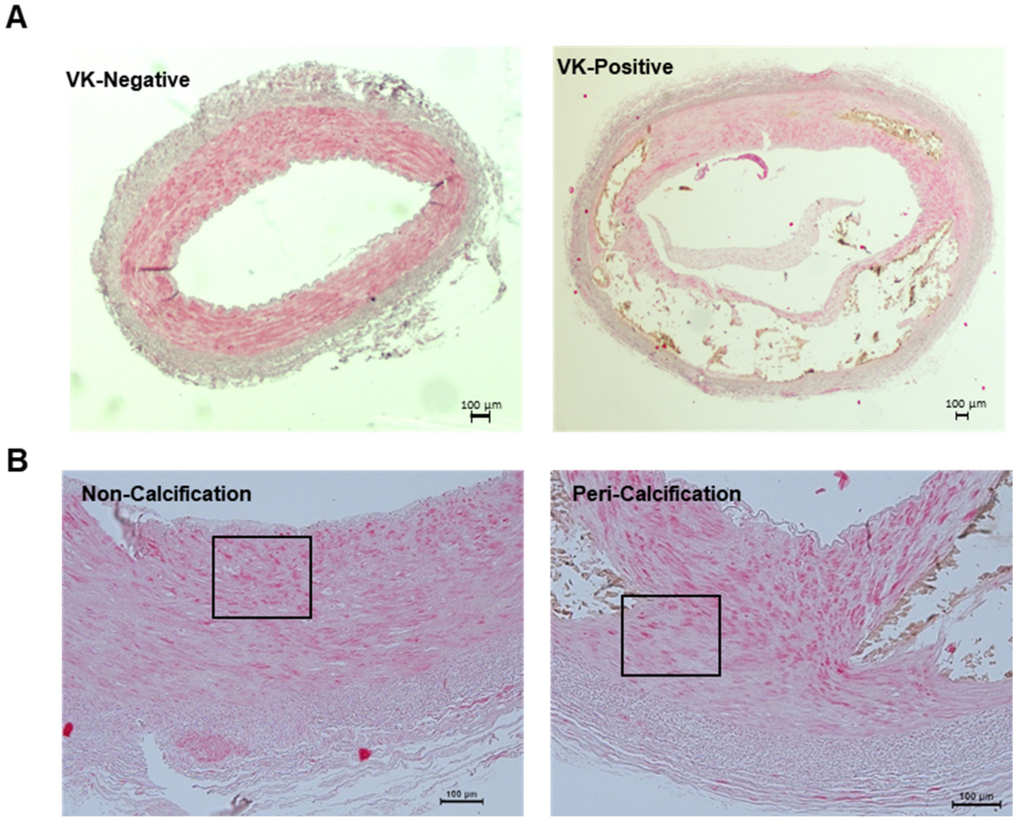

Supplementary Figure S1: Representative images of von Kossa staining. (A) Epigastric arteria negative and positive for von kossa staining. (B) Micrographies of distant and peri-calcification areas of the same epigastric ar-teria section for Von Kossa staining.

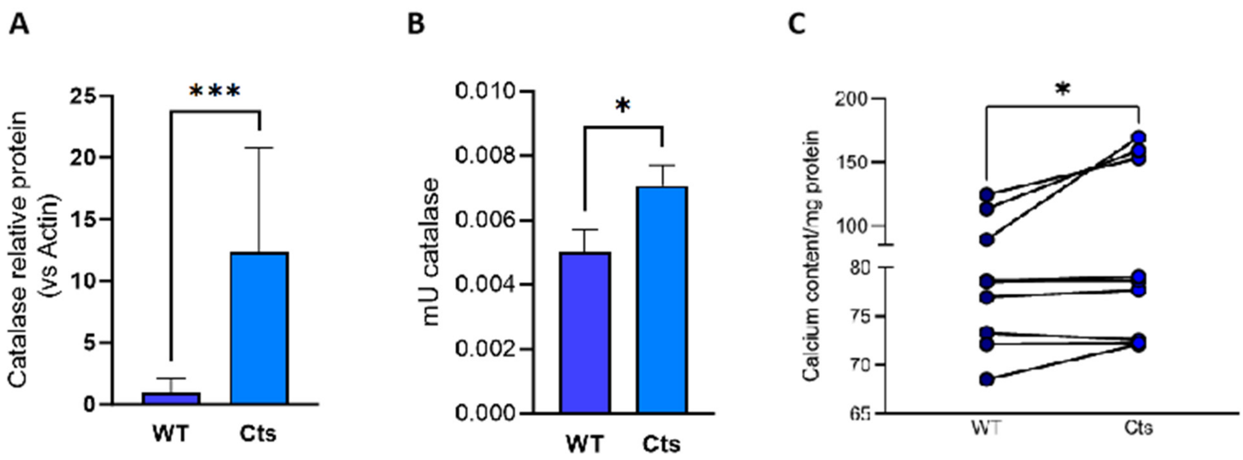

Supplementary Figure S2: VSMCs from Wild-type (WT) and Catalase (Cts) cell culture. (A) Relative optical density of western-blot data for catalase protein expression. (B) Cts activity in VSMC cell culture. (C) Calcium content per mg of protein in WT cell culture and Cts overexpressing cells. * = *p*-value < 0.05; *** = *p*-value < 0.001. (Pools of primary VSMCs were obtained from N=5 wt and N=5 overexpressing catalase mice, 2 independent experiments with 3 replicates were analysed).

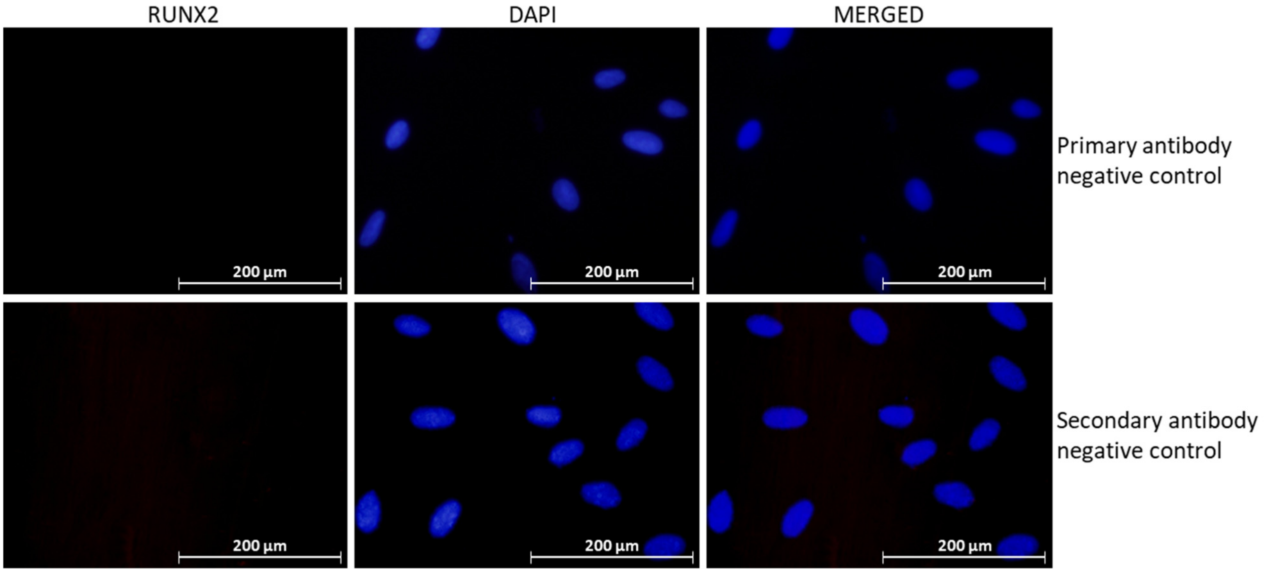

Supplementary Figure S3. Immunofluorescence of RUNX2 in VSMCs. Negative controls for primary (rabbit anti-RUNX2) and secondary antibody (anti-rabbit Alexa Fluor 594). DAPI is used as nuclear counter-staining.

Supplementary Table S1: Biochemical parameters of sham-operated rats with normal renal function without vascular calcification (No-VC) and nephrectomised rats fed a high phosphorus (HPD) with vascular calcification (VC) diet at 20 weeks.

	No-VC (N = 3)			VC (N = 3)			
	**Mean**	**SD**	**95%CI**	**Mean**	**SD**	**95%CI**	**p-value**
**Creatinine (mg/dL)**	0.43	0.05	0.29-0.57	3.2	0.52	1.88-4.51	0.011
**Urea (mg/dL)**	39	13.07	6.52-71.48	262	39.66	163.48-360-52	0.001
**Albumin (mg/dL)**	46.9	1.75	42.54-51.25	29.46	2.77	22.56-36.37	0.001
**Phosphorus (mg/dL)**	5.06	1.19	2.10-8.03	15.16	2.22	9.64-20.69	0.002
**Calcium (mg/dL)**	11.8	0.79	9.82-13.77	10.9	0.10	10.65-11.14	0.187

SD: standard deviation; CI: confidence interval

Supplementary Table S2. Summary of the peptides identified by LC-MS/MS. More detailed information can be found in the annexe.

Spot Nº	MASCOT Symbol	Peptide sequence	Observed m/z	Ion charge	Mr(expt)	Mr(calc)	Delta	Score	Matches	RMS error (ppm)
1	CAH3	GGPLSGPYR	452.4518	2+	902.8890	902.4610	0.4279	50	15/64	399
		VVFDDTFDR	557.5192	2+	1113.0239	1112.5139	0.5100	43	20/72	216
		EPMTVSSDQMAK	678.4788	2+	1354.9431	1354.5745	0.3686	53	53/200	472
2	GPX3	QEPGENSEILPSLK	770.9166	2+	1539.8186	1540.7620	-0.9434	57	31/150	1123
		NSCPPTAELLGSPGR	778.4015	2+	1554.7885	1554.7460	0.0424	68	21/160	1341
3	GSTM2	LYSEFLGK	478.9332	2+	955.8518	955.5015	0.3503	32	10/58	794
		ITQSNAILR	508.4895	2+	1014.9644	1014.5822	0.3822	65	22/80	570
		CLDAFPNLK	539.4450	2+	1076.8755	1076.5325	0.3430	37	22/68	950
		YSMGDAPDYDR	653.3469	2+	1304.6793	1304.4979	0.1814	58	17/140	1019
		VDVLENQAMDTR	703.9233	2+	1405.8321	1405.6507	0.1814	78	41/184	558
		LFLEYTDTSYEDK	812.3617	2+	1622.7088	1622.7352	-0.0264	63	13/112	305
		LFLEYTDTSYEDKK	876.3865	2+	1750.7584	1750.8301	-0.0717	68	21/122	780
4	SODM	GELLEAIKR	514.9566	2+	1027.8986	1027.6026	0.2961	51	20/72	1109
		GDVTTQVALQPALK	720.9116	2+	1439.8087	1439.7984	0.0103	93	20/126	936
5	TKT	LAVSQVPR	435.4305	2+	868.8465	868.5130	0.3335	40	12/62	291
		HQPTAIIAK	489.9728	2+	977.9310	977.5658	0.3652	29	17/78	1219

MASCOT search parameters; Database: SwissProt 2022_04 (accessed 12/14/2022), Taxonomy: Rattus (8,180 sequences), Enzyme: Trypsin, Max missed cleavages: 1, Fixed modifications: Carbamidomethyl (C), Variable modifications: Deaminated (NQ) and Oxidation (M), Peptide mass tolerance: 1.2 Da, Fragment mass tolerance: 0.6 Da, Instrument: ESI-TRAP.

ANNEXE

MS/MS Fragmentation of **GGPLSGPYR** found in **CAH3**

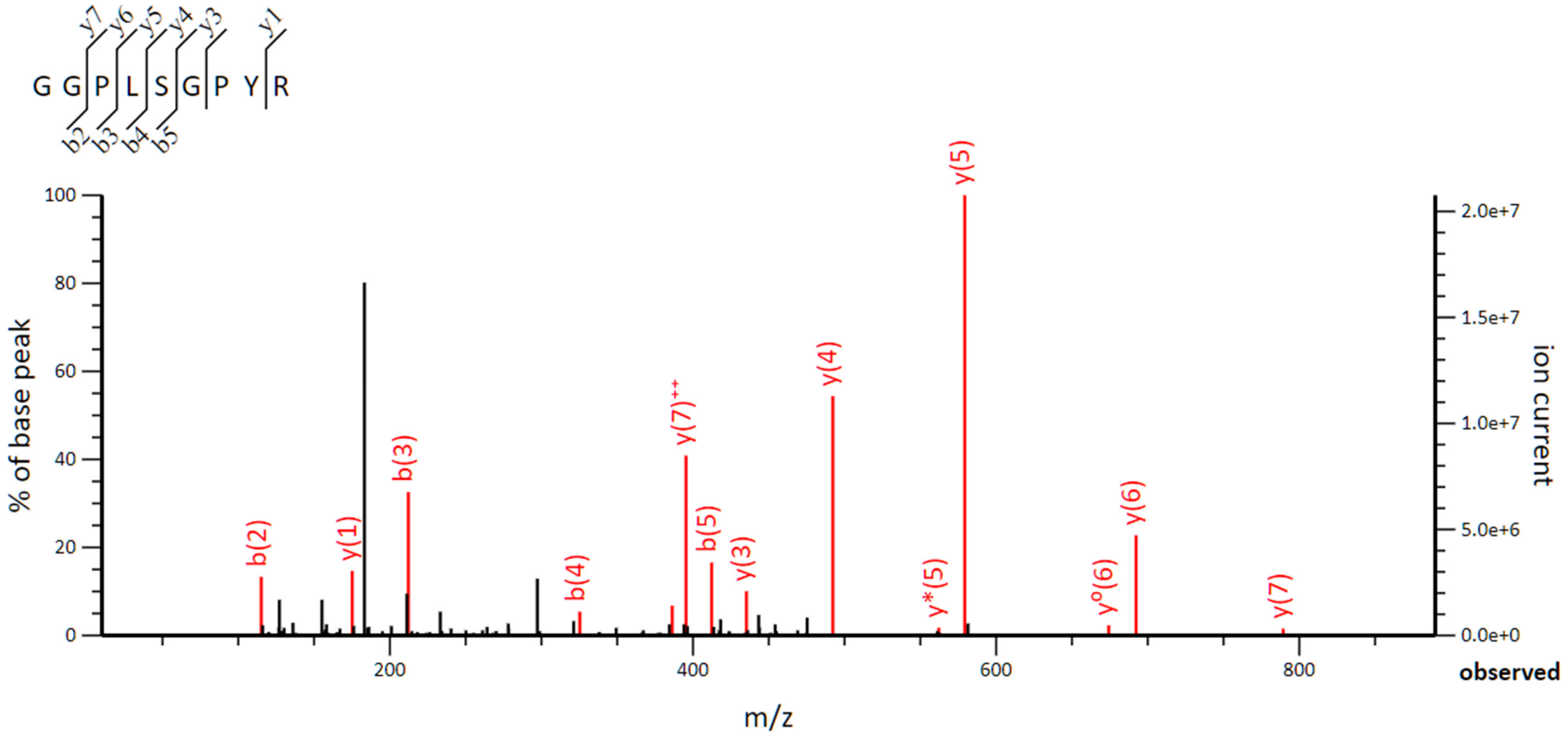

Monoisotopic mass of neutral peptide Mr(calc): 902.4610
Ions Score: 50 **Expect:** 0.00024
Matches: 15/64 fragment ions using 19 most intense peaks

#	b	b++	b0	b0++	Seq.	y	y++	y*	y*++	y0	y0++	#
1	58.0287	29.5180			G							9
2	**115.0502**	58.0287			G	846.4468	423.7271	829.4203	415.2138	828.4363	414.7218	8
3	**212.1030**	106.5551			P	***789.4254***	395.2163	772.3988	386.7030	771.4148	386.2110	7
4	**325.1870**	163.0972			L	***692.3726***	346.6899	675.3461	338.1767	674.3620	337.6847	6
5	**412.2191**	206.6132	394.2085	197.6079	S	***579.2885***	290.1479	562.2620	281.6346	561.2780	281.1426	5
6	469.2405	235.1239	451.2300	226.1186	G	***492.2565***	246.6319	475.2300	238.1186			4
7	566.2933	283.6503	548.2827	274.6450	P	***435.2350***	218.1212	418.2085	209.6079			3
8	729.3566	365.1819	711.3461	356.1767	Y	338.1823	169.5948	321.1557	161.0815			2
9					R	***175.1190***	88.0631	158.0924	79.5498			1

MS/MS Fragmentation of **VVFDDTFDR** found in **CAH3**

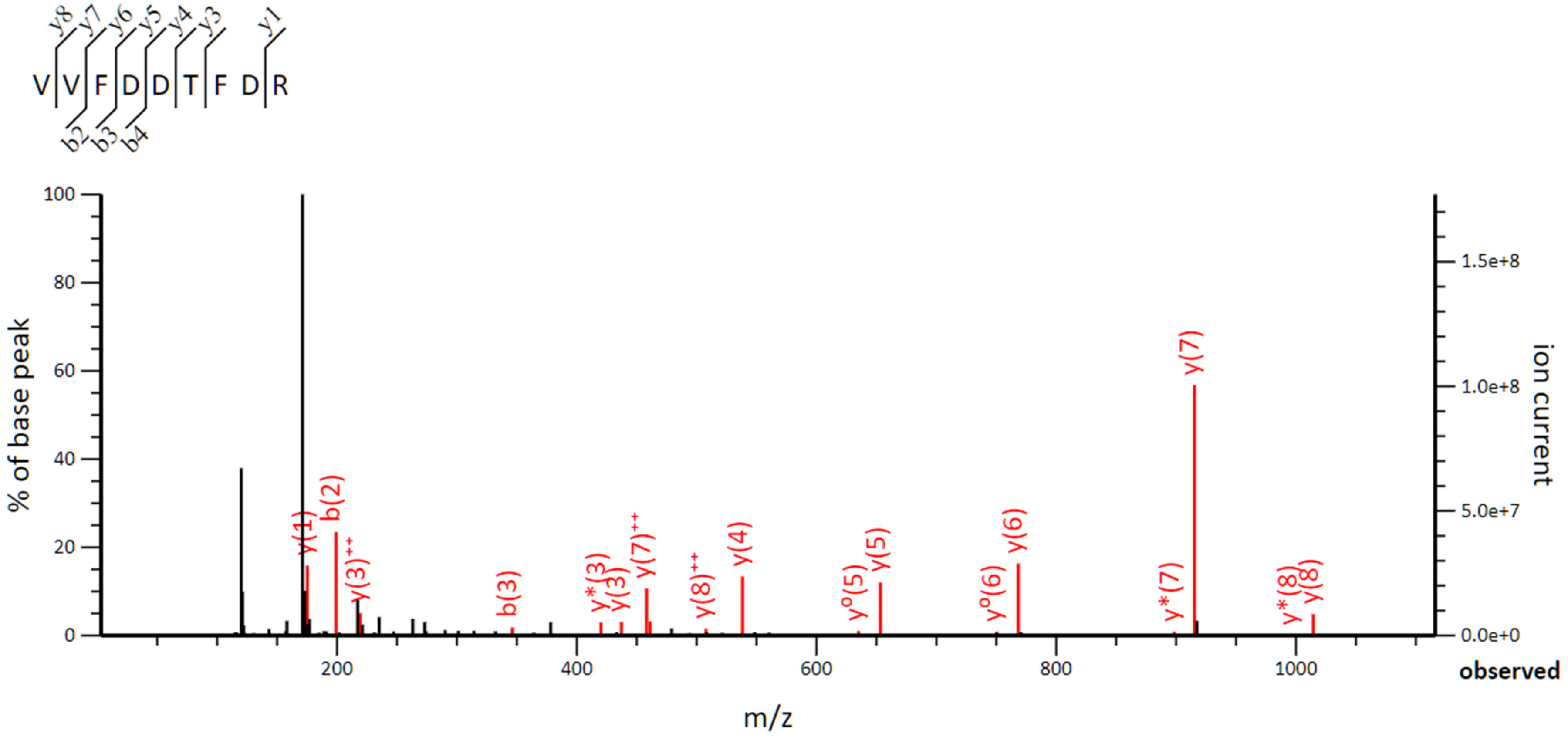

Monoisotopic mass of neutral peptide Mr(calc): 1112.5139
Ions Score: 43 **Expect:** 0.0017
Matches: 20/72 fragment ions using 38 most intense peaks

#	b	b^{++}	b^{0}	b^{0++}	Seq.	y	y^{++}	y*	y^{*++}	y^{0}	y^{0++}	#
1	100.0757	50.5415			V							9
2	**199.1441**	100.0757			V	***1014.4527***	**507.7300**	997.4262	499.2167	996.4421	498.7247	8
3	**346.2125**	173.6099			F	***915.3843***	**458.1958**	898.3577	449.6825	897.3737	449.1905	7
4	**461.2395**	231.1234	443.2289	222.1181	D	***768.3159***	384.6616	751.2893	376.1483	750.3053	375.6563	6
5	576.2664	288.6368	558.2558	279.6316	D	***653.2889***	327.1481	636.2624	318.6348	635.2784	318.1428	5
6	677.3141	339.1607	659.3035	330.1554	T	***538.2620***	269.6346	521.2354	261.1214	520.2514	260.6293	4
7	824.3825	412.6949	806.3719	403.6896	F	***437.2143***	**219.1108**	420.1878	210.5975	419.2037	210.1055	3
8	939.4094	470.2084	921.3989	461.2031	D	290.1459	145.5766	273.1193	137.0633	272.1353	136.5713	2
9					R	***175.1190***	88.0631	158.0924	79.5498			1

MS/MS Fragmentation of **EPMTVSSDQMAK** found in **CAH3**

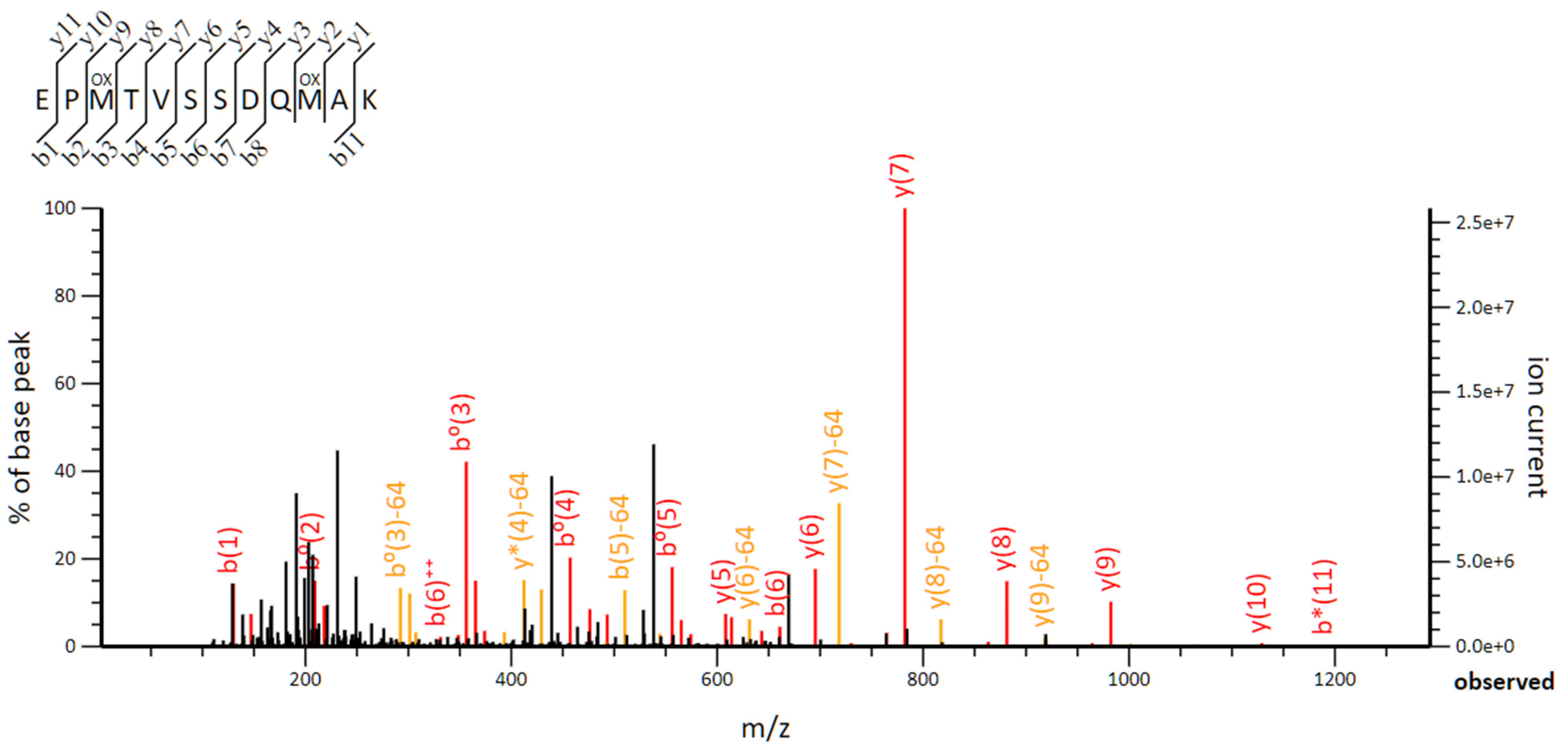

Monoisotopic mass of neutral peptide Mr(calc): 1354.5745
Ions Score: 53 **Expect:** 4.1e-05
Matches : 53/200 fragment ions using 84 most intense peaks

#	b	b^{++}	b*	b^{*++}	b^0	b^{0++}	Seq.	y	y^{++}	y*	y^{*++}	y^0	y^{0++}	#
1	**130.0499**	65.5286			112.0393	56.5233	E							12
2	227.1026	114.0550			209.0921	105.0497	P	1226.5392	**613.7732**	1209.5126	605.2599	1208.5286	604.7679	11
3	**374.1380**	187.5727			356.1275	178.5674	M	***1129.4864***	**565.2468**	1112.4598	556.7336	1111.4758	556.2415	10
4	475.1857	238.0965			457.1751	229.0912	T	***982.4510***	491.7291	965.4244	483.2159	964.4404	482.7238	9
5	**574.2541**	287.6307			556.2436	278.6254	V	***881.4033***	441.2053	864.3768	432.6920	863.3927	432.2000	8
6	**661.2862**	331.1467			643.2756	322.1414	S	***782.3349***	391.6711	765.3083	383.1578	764.3243	382.6658	7
7	748.3182	374.6627			730.3076	365.6574	S	***695.3029***	**348.1551**	678.2763	339.6418	677.2923	339.1498	6
8	**863.3451**	432.1762			845.3346	423.1709	D	***608.2708***	304.6391	591.2443	296.1258	590.2603	295.6338	5
9	991.4037	496.2055	974.3772	487.6922	973.3931	487.2002	Q	***493.2439***	247.1256	476.2173	238.6123			4
10	1138.4391	569.7232	1121.4126	561.2099	1120.4285	560.7179	M	***365.1853***	183.0963	348.1588	174.5830			3
11	1209.4762	605.2417	1192.4497	596.7285	1191.4657	596.2365	A	***218.1499***	109.5786	201.1234	101.0653			2
12							K	***147.1128***	74.0600	130.0863	65.5468			1

MS/MS Fragmentation of **QEPGENSEILPSLK** found in **GPX3**

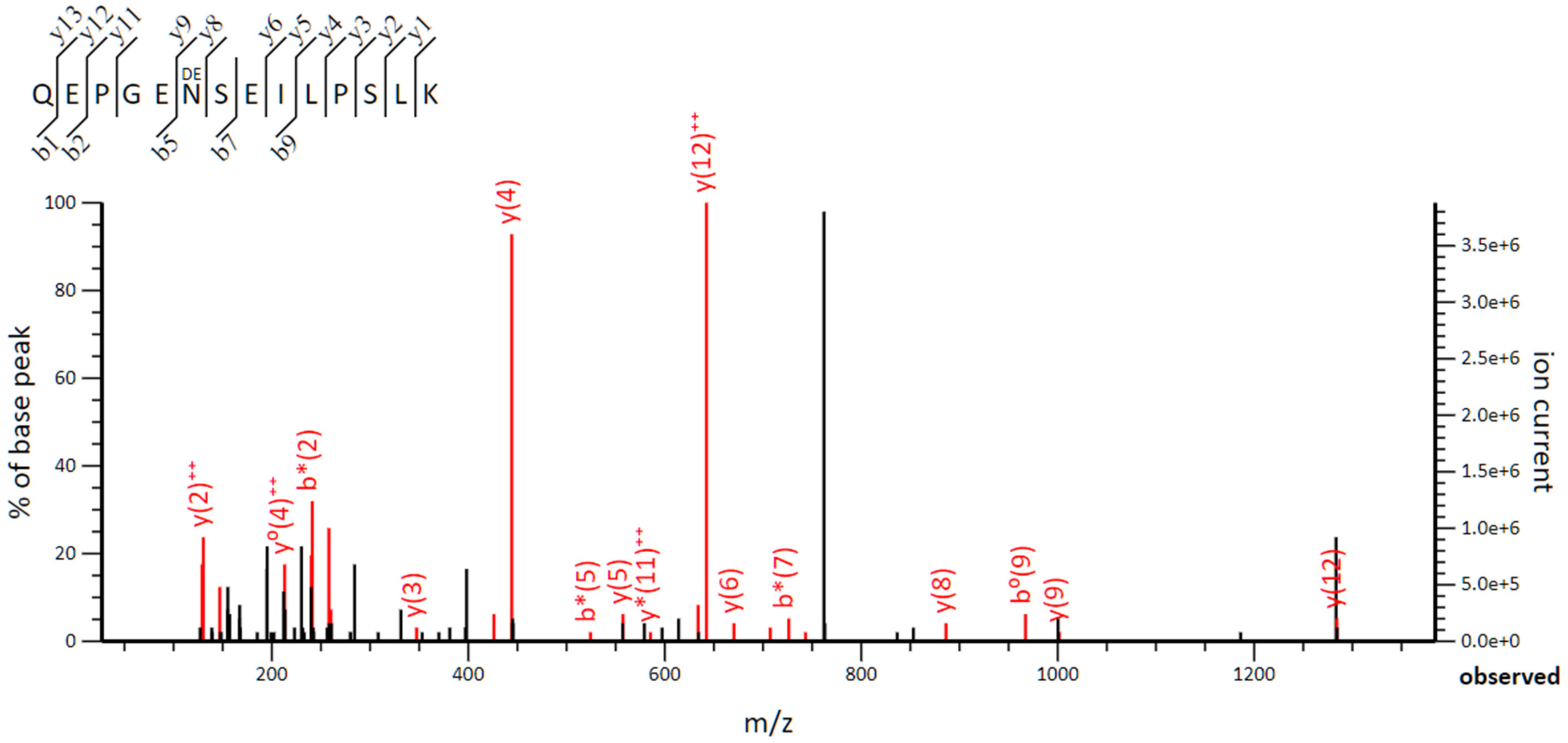

Monoisotopic mass of neutral peptide Mr(calc): 1540.7620
Ions Score: 57 **Expect:** 0.00014
Matches : 31/150 fragment ions using 51 most intense peaks

#	b	b^{++}	b*	b^{*++}	b^0	b^{0++}	Seq.	y	y^{++}	y*	y^{*++}	y^0	y^{0++}	#
1	**129.0659**	65.0366	112.0393	56.5233			Q							14
2	**258.1084**	129.5579	241.0819	121.0446	240.0979	120.5526	E	1413.7108	**707.3590**	1396.6842	698.8457	1395.7002	698.3537	13
3	355.1612	178.0842	338.1347	169.5710	337.1506	169.0790	P	***1284.6682***	**642.8377**	1267.6416	634.3244	1266.6576	633.8324	12
4	412.1827	206.5950	395.1561	198.0817	394.1721	197.5897	G	1187.6154	594.3113	1170.5889	585.7981	1169.6048	585.3061	11
5	541.2253	271.1163	524.1987	262.6030	523.2147	262.1110	E	1130.5939	565.8006	1113.5674	557.2873	1112.5834	556.7953	10
6	656.2522	328.6297	639.2257	320.1165	638.2416	319.6245	N	***1001.5514***	501.2793	984.5248	492.7660	983.5408	492.2740	9
7	**743.2842**	372.1458	726.2577	363.6325	725.2737	363.1405	S	***886.5244***	**443.7658**	869.4979	435.2526	868.5138	434.7606	8
8	872.3268	436.6671	855.3003	428.1538	854.3163	427.6618	E	799.4924	400.2498	782.4658	391.7366	781.4818	391.2445	7
9	985.4109	493.2091	968.3843	484.6958	967.4003	484.2038	I	***670.4498***	335.7285	653.4232	327.2153	652.4392	326.7232	6
10	1098.4950	549.7511	1081.4684	541.2378	1080.4844	540.7458	L	***557.3657***	279.1865	540.3392	270.6732	539.3552	270.1812	5
11	1195.5477	598.2775	1178.5212	589.7642	1177.5372	589.2722	P	***444.2817***	222.6445	427.2551	214.1312	426.2711	213.6392	4
12	1282.5798	641.7935	1265.5532	633.2802	1264.5692	632.7882	S	***347.2289***	174.1181	330.2023	165.6048	329.2183	165.1128	3
13	1395.6638	698.3355	1378.6373	689.8223	1377.6533	689.3303	L	***260.1969***	**130.6021**	243.1703	122.0888			2
14							K	***147.1128***	74.0600	130.0863	65.5468			1

MS/MS Fragmentation of **NSCPPTAELLGSPGR** found in **GPX3**

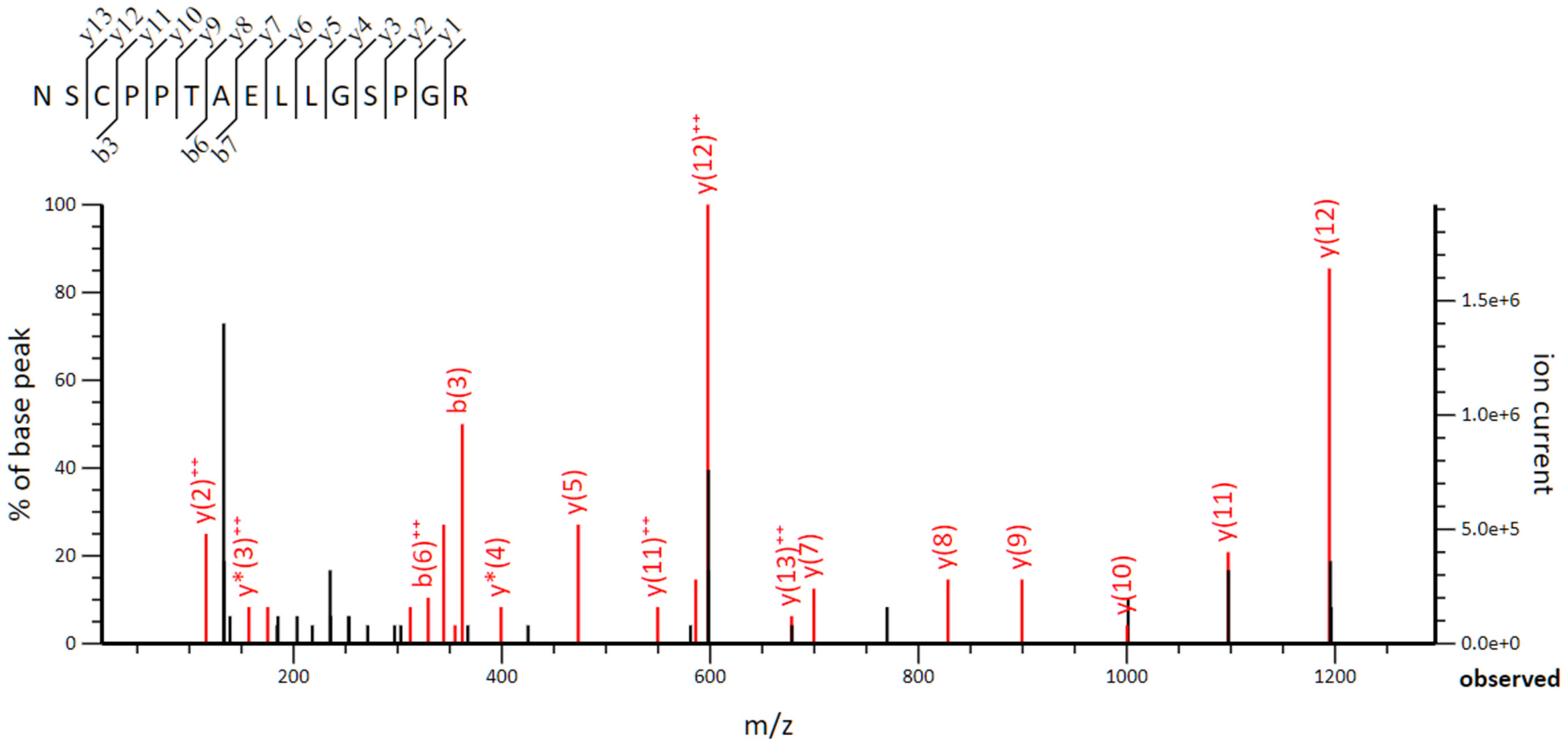

Monoisotopic mass of neutral peptide Mr(calc): 1554.7460
Ions Score: 68 **Expect:** 2.2e-06
Matches : 21/160 fragment ions using 35 most intense peaks

#	b	b++	b*	b*++	b0	b0++	Seq.	y	y++	y*	y*++	y0	y0++	#
1	115.0502	58.0287	98.0237	49.5155			N							15
2	202.0822	101.5448	185.0557	93.0315	184.0717	92.5395	S	1441.7104	721.3588	1424.6838	712.8456	1423.6998	712.3536	14
3	362.1129	181.5601	345.0863	173.0468	344.1023	172.5548	C	1354.6784	**677.8428**	1337.6518	669.3295	1336.6678	668.8375	13
4	459.1656	230.0865	442.1391	221.5732	441.1551	221.0812	P	***1194.6477***	**597.8275**	1177.6212	589.3142	1176.6371	588.8222	12
5	556.2184	278.6128	539.1919	270.0996	538.2078	269.6076	P	***1097.5949***	**549.3011**	1080.5684	540.7878	1079.5844	540.2958	11
6	657.2661	329.1367	640.2395	320.6234	639.2555	320.1314	T	***1000.5422***	500.7747	983.5156	492.2615	982.5316	491.7694	10
7	728.3032	364.6552	711.2767	356.1420	710.2926	355.6500	A	***899.4945***	450.2509	882.4680	441.7376	881.4839	441.2456	9
8	857.3458	429.1765	840.3192	420.6633	839.3352	420.1713	E	***828.4574***	414.7323	811.4308	406.2191	810.4468	405.7271	8
9	970.4299	485.7186	953.4033	477.2053	952.4193	476.7133	L	***699.4148***	350.2110	682.3883	341.6978	681.4042	341.2058	7
10	1083.5139	542.2606	1066.4874	533.7473	1065.5034	533.2553	L	***586.3307***	293.6690	569.3042	285.1557	568.3202	284.6637	6
11	1140.5354	570.7713	1123.5088	562.2581	1122.5248	561.7660	G	***473.2467***	237.1270	456.2201	228.6137	455.2361	228.1217	5
12	1227.5674	614.2873	1210.5409	605.7741	1209.5568	605.2821	S	416.2252	208.6162	399.1987	200.1030	398.2146	199.6110	4
13	1324.6202	662.8137	1307.5936	654.3005	1306.6096	653.8084	P	***329.1932***	165.1002	312.1666	156.5870			3
14	1381.6416	691.3245	1364.6151	682.8112	1363.6311	682.3192	G	232.1404	**116.5738**	215.1139	108.0606			2
15							R	***175.1190***	88.0631	158.0924	79.5498			1

MS/MS Fragmentation of **LYSEFLGK** found in **GSTM2**

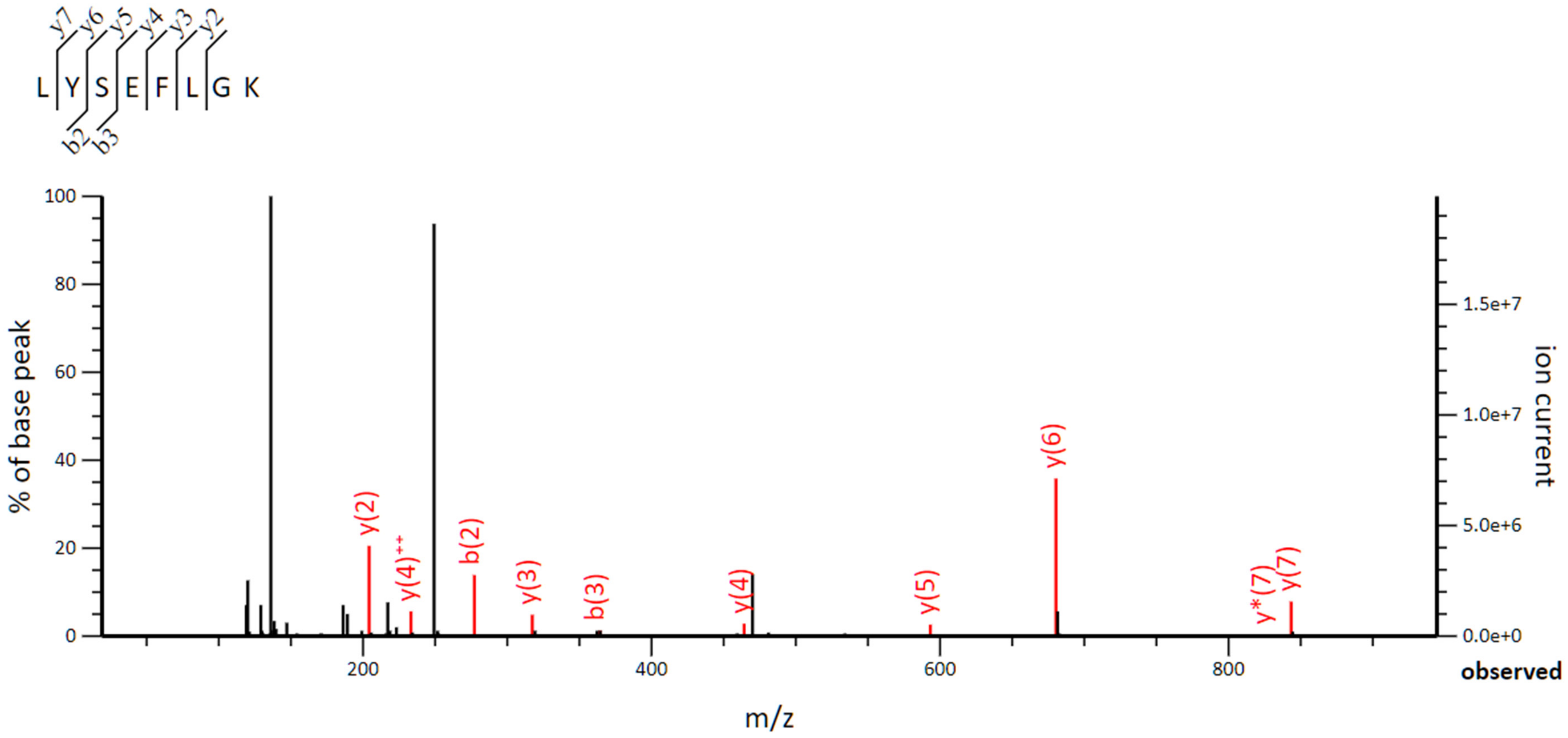

Monoisotopic mass of neutral peptide Mr(calc): 955.5015
Ions Score: 32 **Expect:** 0.0063
Matches : 10/58 fragment ions using 24 most intense peaks

#	b	b^{++}	b^0	b^{0++}	Seq.	y	y^{++}	y*	y^{*++}	y^0	y^{0++}	#
1	114.0913	57.5493			L							8
2	**277.1547**	139.0810			Y	***843.4247***	422.2160	826.3981	413.7027	825.4141	413.2107	7
3	**364.1867**	182.5970	346.1761	173.5917	S	***680.3614***	340.6843	663.3348	332.1710	662.3508	331.6790	6
4	493.2293	247.1183	475.2187	238.1130	E	***593.3293***	297.1683	576.3028	288.6550	575.3188	288.1630	5
5	640.2977	320.6525	622.2871	311.6472	F	***464.2867***	232.6470	447.2602	224.1337			4
6	753.3818	377.1945	735.3712	368.1892	L	***317.2183***	159.1128	300.1918	150.5995			3
7	810.4032	405.7053	792.3927	396.7000	G	***204.1343***	102.5708	187.1077	94.0575			2
8					K	147.1128	74.0600	130.0863	65.5468			1

MS/MS Fragmentation of **ITQSNAILR** found in **GSTM2**

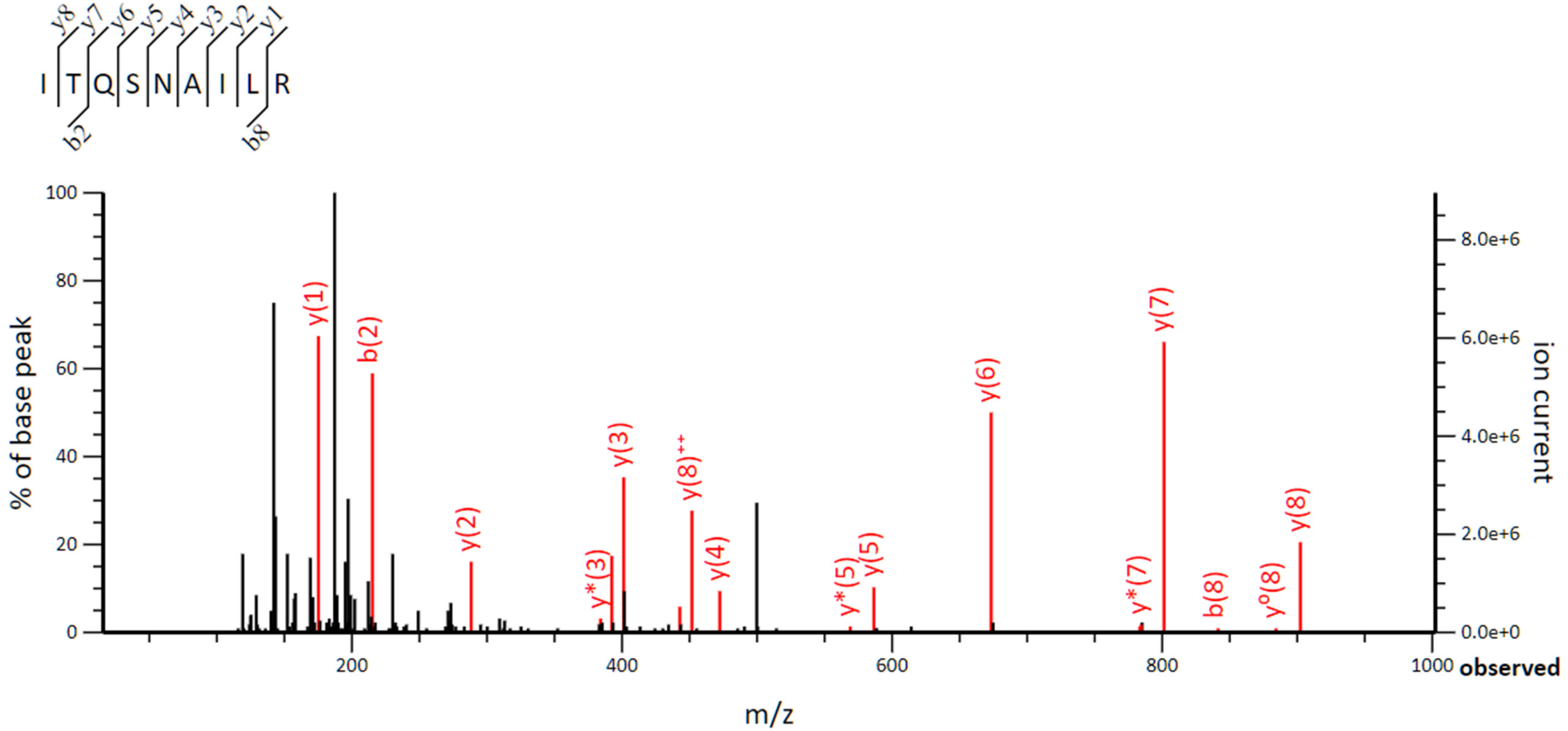

Monoisotopic mass of neutral peptide Mr(calc): 1014.5822
Ions Score: 65 **Expect:** 2.3e-05
Matches : 22/80 fragment ions using 29 most intense peaks

#	b	b++	b*	b*++	b0	b0++	Seq.	y	y++	y*	y*++	y0	y0++	#
1	114.0913	57.5493					I							9
2	**215.1390**	108.0731			197.1285	99.0679	T	***902.5054***	**451.7563**	885.4789	443.2431	884.4948	442.7511	8
3	343.1976	172.1024	326.1710	163.5892	325.1870	163.0972	Q	***801.4577***	**401.2325**	784.4312	392.7192	783.4472	392.2272	7
4	430.2296	215.6185	413.2031	207.1052	412.2191	206.6132	S	***673.3991***	337.2032	656.3726	328.6899	655.3886	328.1979	6
5	544.2726	272.6399	527.2460	264.1266	526.2620	263.6346	N	***586.3671***	293.6872	569.3406	285.1739			5
6	615.3097	308.1585	598.2831	299.6452	597.2991	299.1532	A	***472.3242***	236.6657	455.2976	228.1525			4
7	728.3937	364.7005	711.3672	356.1872	710.3832	355.6952	I	***401.2871***	201.1472	384.2605	192.6339			3
8	**841.4778**	421.2425	824.4512	412.7293	823.4672	412.2373	L	***288.2030***	144.6051	271.1765	136.0919			2
9							R	***175.1190***	88.0631	158.0924	79.5498			1

MS/MS Fragmentation of **CLDAFPNLK** found in **GSTM2**

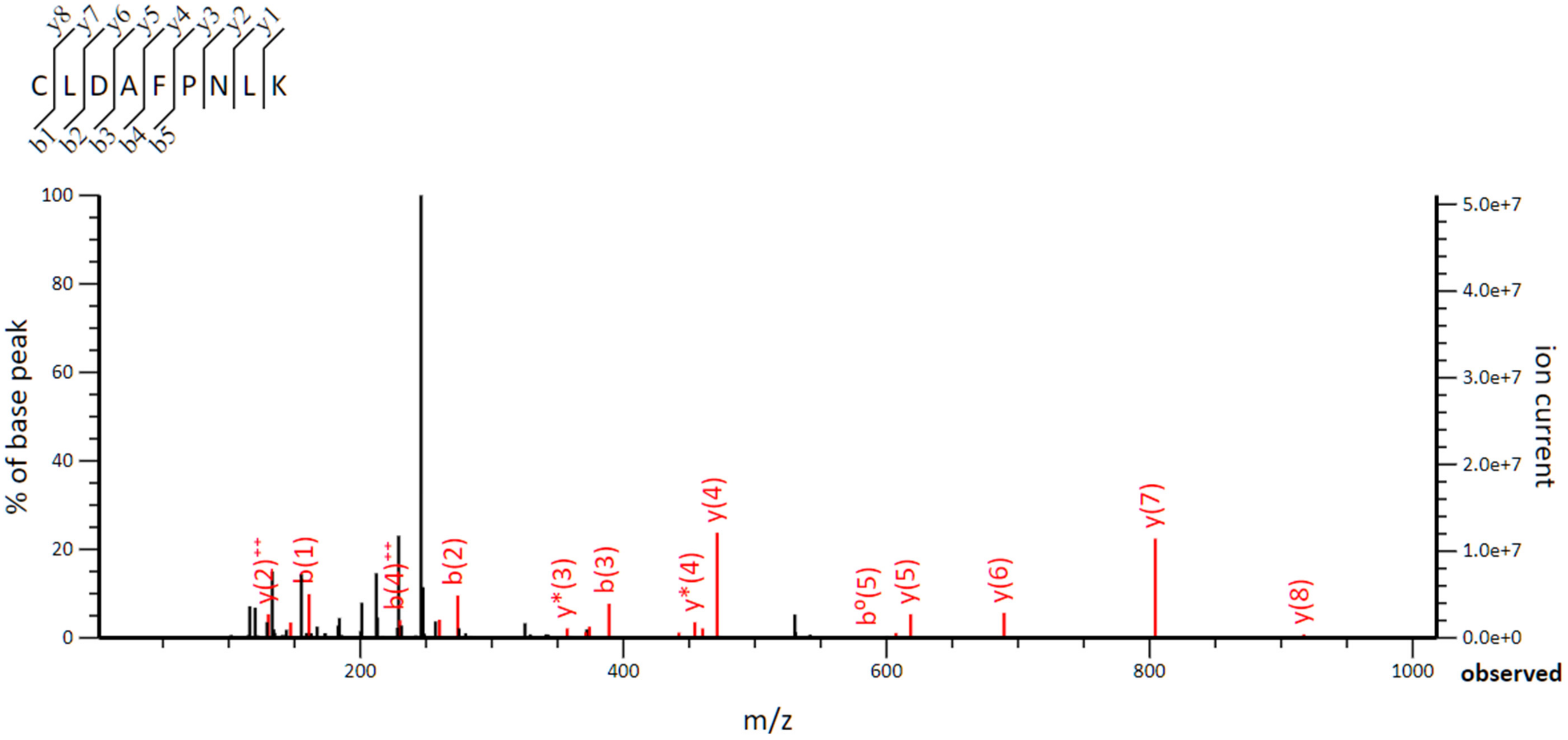

Monoisotopic mass of neutral peptide Mr(calc): 1076.5325
Ions Score: 37 **Expect:** 0.0037
Matches : 22/68 fragment ions using 62 most intense peaks

#	b	b^{++}	b^{*}	b^{*++}	b^{0}	b^{0++}	Seq.	y	y^{++}	y^{*}	y^{*++}	y^{0}	y^{0++}	#
1	**161.0379**	81.0226					C							9
2	**274.1220**	137.5646					L	***917.5091***	459.2582	900.4825	450.7449	899.4985	450.2529	8
3	**389.1489**	195.0781			371.1384	186.0728	D	***804.4250***	402.7162	787.3985	394.2029	786.4145	393.7109	7
4	**460.1860**	230.5967			442.1755	221.5914	A	***689.3981***	345.2027	672.3715	336.6894			6
5	**607.2545**	304.1309			589.2439	295.1256	F	***618.3610***	309.6841	601.3344	301.1708			5
6	704.3072	352.6573			686.2967	343.6520	P	***471.2926***	236.1499	454.2660	227.6366			4
7	818.3502	409.6787	801.3236	401.1654	800.3396	400.6734	N	***374.2398***	187.6235	357.2132	179.1103			3
8	931.4342	466.2207	914.4077	457.7075	913.4237	457.2155	L	***260.1969***	130.6021	243.1703	122.0888			2
9							K	***147.1128***	74.0600	130.0863	65.5468			1

MS/MS Fragmentation of **YSMGDAPDYDR** found in **GSTM2**

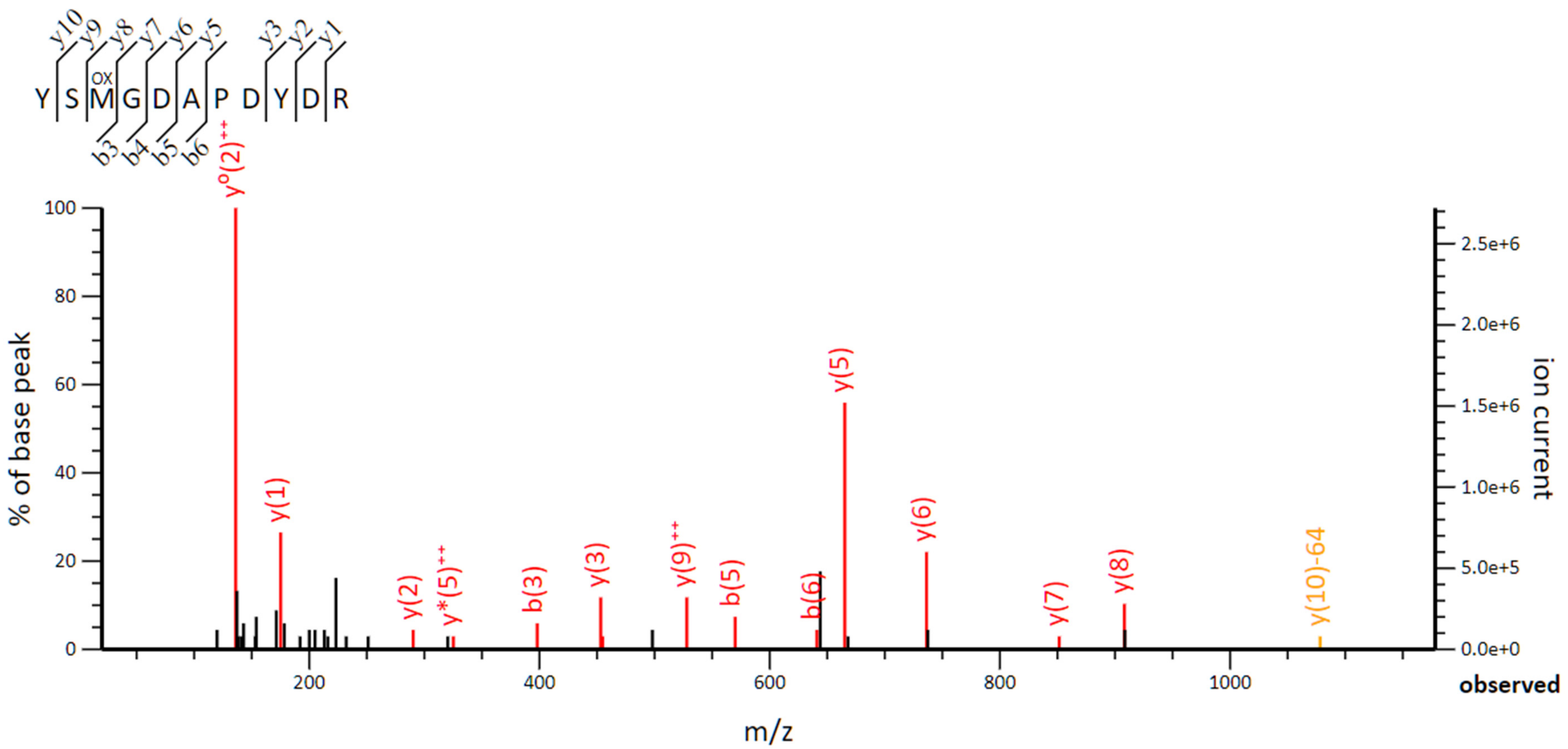

Monoisotopic mass of neutral peptide Mr(calc): 1304.4979
Ions Score: 58 **Expect:** 0.00016
Matches : 17/140 fragment ions using 23 most intense peaks

#	b	b++	b0	b0++	Seq.	y	y++	y*	y*++	y0	y0++	#
1	164.0706	82.5389			Y							11
2	251.1026	126.0550	233.0921	117.0497	S	1142.4419	571.7246	1125.4153	563.2113	1124.4313	562.7193	10
3	**398.1380**	199.5727	380.1275	190.5674	M	1055.4099	**528.2086**	1038.3833	519.6953	1037.3993	519.2033	9
4	**455.1595**	228.0834	437.1489	219.0781	G	***908.3745***	**454.6909**	891.3479	446.1776	890.3639	445.6856	8
5	**570.1864**	285.5969	552.1759	276.5916	D	***851.3530***	426.1801	834.3264	417.6669	833.3424	417.1748	7
6	**641.2236**	321.1154	623.2130	312.1101	A	***736.3260***	368.6667	719.2995	360.1534	718.3155	359.6614	6
7	738.2763	369.6418	720.2658	360.6365	P	***665.2889***	333.1481	648.2624	324.6348	647.2784	324.1428	5
8	853.3033	427.1553	835.2927	418.1500	D	568.2362	284.6217	551.2096	276.1084	550.2256	275.6164	4
9	1016.3666	508.6869	998.3560	499.6817	Y	***453.2092***	227.1082	436.1827	218.5950	435.1987	218.1030	3
10	1131.3935	566.2004	1113.3830	557.1951	D	***290.1459***	145.5766	273.1193	137.0633	272.1353	136.5713	2
11					R	***175.1190***	88.0631	158.0924	79.5498			1

MS/MS Fragmentation of **VDVLENQAMDTR** found in **GSTM2**

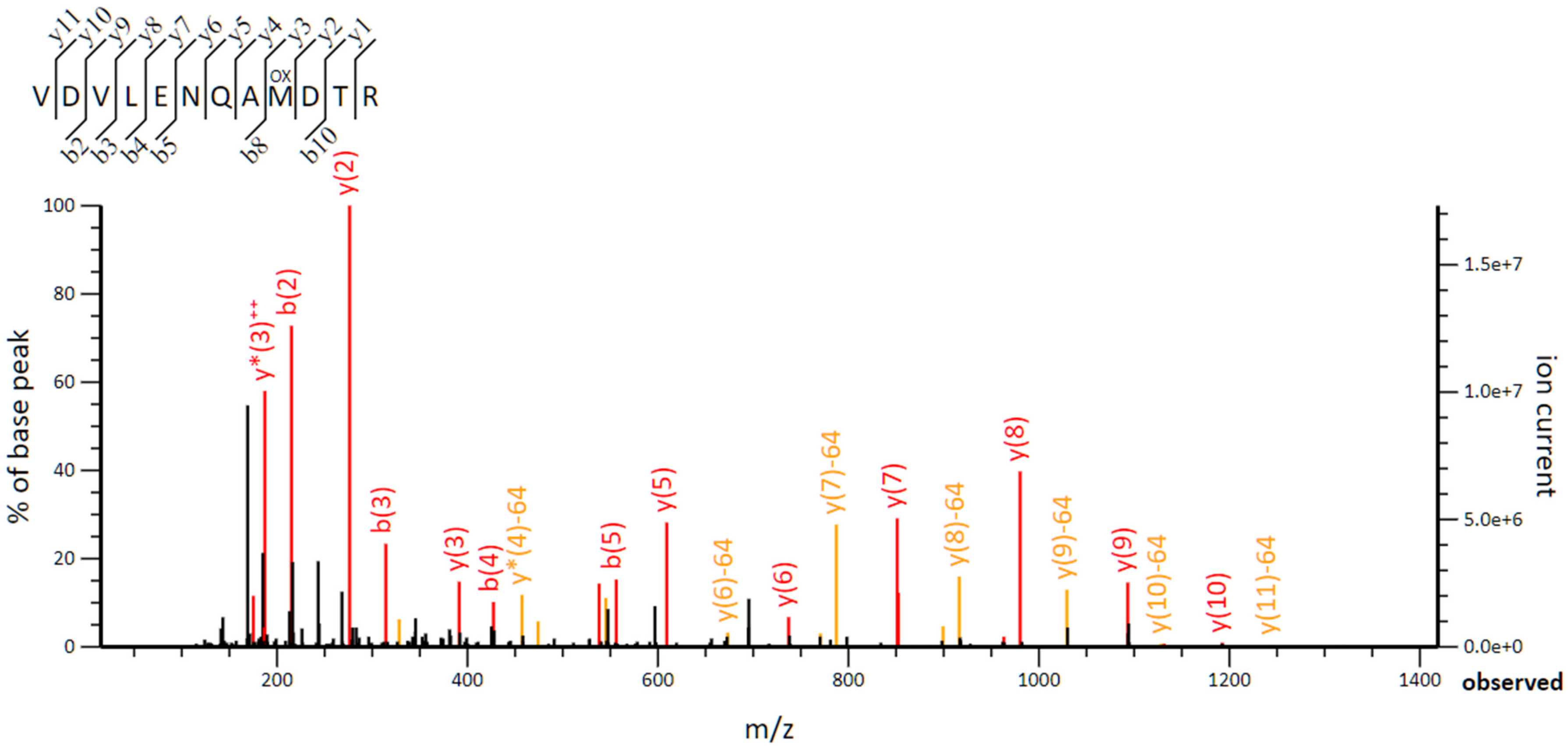

Monoisotopic mass of neutral peptide Mr(calc): 1405.6507
Ions Score: 78 **Expect:** 1.7e-06
Matches : 41/184 fragment ions using 46 most intense peaks

#	b	b++	b*	b*++	b0	b0++	Seq.	y	y++	y*	y*++	y0	y0++	#
1	100.0757	50.5415					V							12
2	**215.1026**	108.0550			197.0921	99.0497	D	***1243.5913***	622.2993	1226.5648	613.7860	1225.5808	613.2940	11
3	**314.1710**	157.5892			296.1605	148.5839	V	***1128.5644***	564.7858	1111.5378	556.2726	1110.5538	555.7805	10
4	**427.2551**	214.1312			409.2445	205.1259	L	***1029.4960***	515.2516	1012.4694	506.7383	1011.4854	506.2463	9
5	**556.2977**	278.6525			538.2871	269.6472	E	***916.4119***	458.7096	899.3854	450.1963	898.4013	449.7043	8
6	670.3406	335.6740	653.3141	327.1607	652.3301	326.6687	N	***787.3693***	394.1883	770.3428	385.6750	769.3587	385.1830	7
7	798.3992	399.7032	781.3727	391.1900	780.3886	390.6980	Q	***673.3264***	337.1668	656.2998	328.6536	655.3158	328.1615	6
8	869.4363	435.2218	852.4098	426.7085	851.4258	426.2165	A	***545.2678***	273.1375	528.2413	264.6243	527.2572	264.1323	5
9	952.4734	476.7404	935.4469	468.2271	934.4629	467.7351	M	***474.2307***	237.6190	457.2041	229.1057	456.2201	228.6137	4
10	1067.5004	534.2538	1050.4738	525.7406	1049.4898	525.2485	D	***391.1936***	196.1004	374.1670	187.5872	373.1830	187.0951	3
11	1168.5481	584.7777	1151.5215	576.2644	1150.5375	575.7724	T	***276.1666***	138.5870	259.1401	130.0737	258.1561	129.5817	2
12							R	***175.1190***	88.0631	158.0924	79.5498			1

MS/MS Fragmentation of **LFLEYTDTSYEDK** found in **GSTM2**

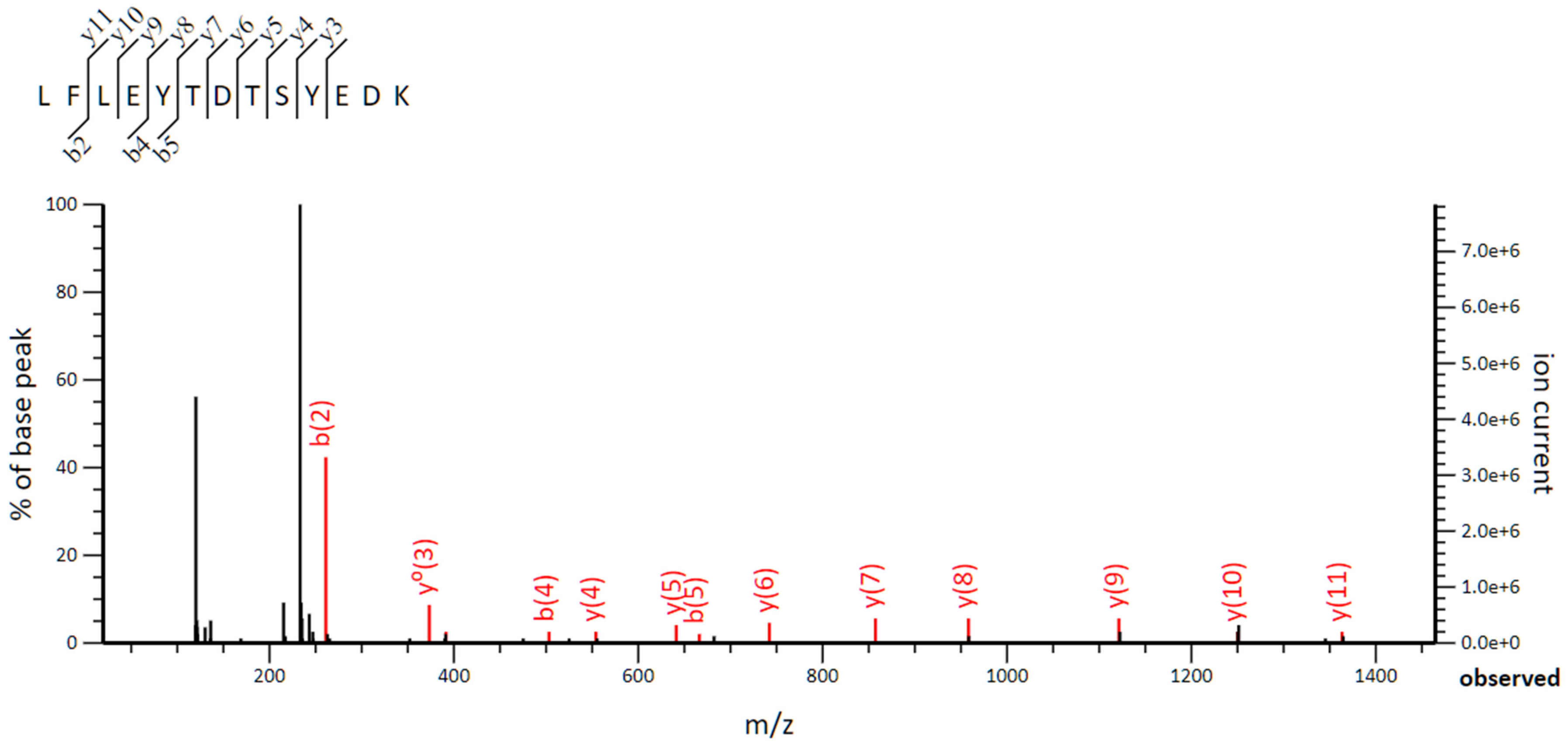

Monoisotopic mass of neutral peptide Mr(calc): 1622.7352
Ions Score: 63 **Expect:** 1.3e-05
Matches : 13/112 fragment ions using 22 most intense peaks

#	b	b^{++}	b^{0}	b^{0++}	Seq.	y	y^{++}	y*	y^{*++}	y^{0}	y^{0++}	#
1	114.0913	57.5493			L							13
2	**261.1598**	131.0835			F	1510.6584	755.8328	1493.6318	747.3196	1492.6478	746.8276	12
3	374.2438	187.6255			L	***1363.5900***	682.2986	1346.5634	673.7854	1345.5794	673.2933	11
4	**503.2864**	252.1468	485.2758	243.1416	E	***1250.5059***	625.7566	1233.4794	617.2433	1232.4954	616.7513	10
5	**666.3497**	333.6785	648.3392	324.6732	Y	***1121.4633***	561.2353	1104.4368	552.7220	1103.4528	552.2300	9
6	767.3974	384.2023	749.3869	375.1971	T	***958.4000***	479.7036	941.3734	471.1904	940.3894	470.6984	8
7	882.4244	441.7158	864.4138	432.7105	D	***857.3523***	429.1798	840.3258	420.6665	839.3418	420.1745	7
8	983.4720	492.2397	965.4615	483.2344	T	***742.3254***	371.6663	725.2988	363.1531	724.3148	362.6610	6
9	1070.5041	535.7557	1052.4935	526.7504	S	***641.2777***	321.1425	624.2511	312.6292	623.2671	312.1372	5
10	1233.5674	617.2873	1215.5568	608.2821	Y	***554.2457***	277.6265	537.2191	269.1132	536.2351	268.6212	4
11	1362.6100	681.8086	1344.5994	672.8034	E	***391.1823***	196.0948	374.1558	187.5815	373.1718	187.0895	3
12	1477.6369	739.3221	1459.6264	730.3168	D	262.1397	131.5735	245.1132	123.0602	244.1292	122.5682	2
13					K	147.1128	74.0600	130.0863	65.5468			1

MS/MS Fragmentation of **LFLEYTDTSYEDKK** found in **GSTM2**

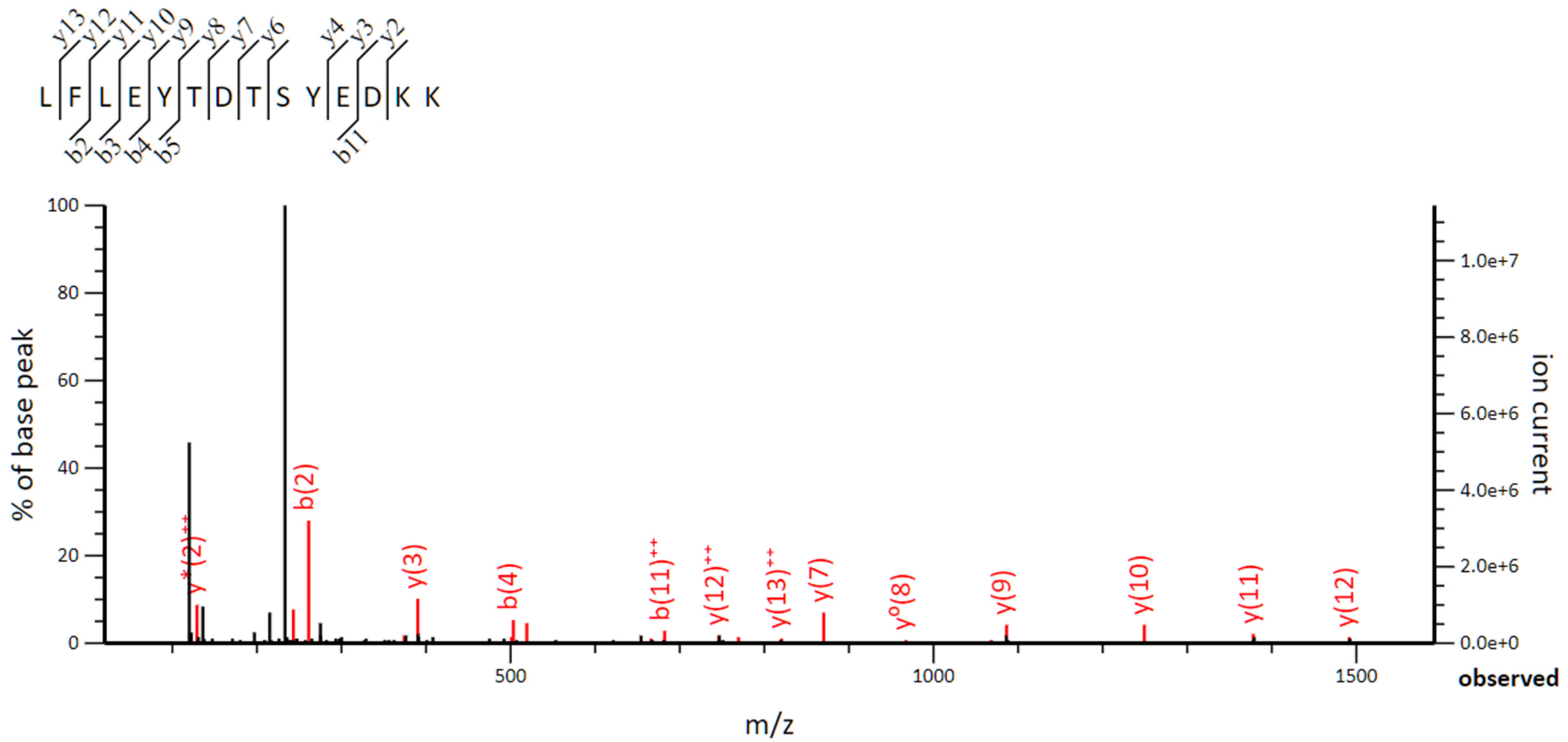

Monoisotopic mass of neutral peptide Mr(calc): 1750.8301
Fixed modifications: Carbamidomethyl (C) (apply to specified residues or termini only)**Ions Score:** 68 **Expect:** 2.9e-06
Matches : 21/122 fragment ions using 31 most intense peaks

#	b	b++	b*	b*++	b0	b0++	Seq.	y	y++	y*	y*++	y0	y0++	#
1	114.0913	57.5493					L							14
2	**261.1598**	131.0835					F	1638.7534	**819.8803**	1621.7268	811.3670	1620.7428	810.8750	13
3	**374.2438**	187.6255					L	***1491.6849***	**746.3461**	1474.6584	737.8328	1473.6744	737.3408	12
4	**503.2864**	252.1468			485.2758	243.1416	E	***1378.6009***	689.8041	1361.5743	681.2908	1360.5903	680.7988	11
5	**666.3497**	333.6785			648.3392	324.6732	Y	***1249.5583***	625.2828	1232.5317	616.7695	1231.5477	616.2775	10
6	767.3974	384.2023			749.3869	375.1971	T	***1086.4950***	543.7511	1069.4684	535.2378	1068.4844	534.7458	9
7	882.4244	441.7158			864.4138	432.7105	D	985.4473	493.2273	968.4207	484.7140	967.4367	484.2220	8
8	983.4720	492.2397			965.4615	483.2344	T	***870.4203***	435.7138	853.3938	427.2005	852.4098	426.7085	7
9	1070.5041	535.7557			1052.4935	526.7504	S	***769.3727***	385.1900	752.3461	376.6767	751.3621	376.1847	6
10	1233.5674	617.2873			1215.5568	608.2821	Y	***682.3406***	341.6740	665.3141	333.1607	664.3301	332.6687	5
11	1362.6100	681.8086			1344.5994	672.8034	E	***519.2773***	260.1423	502.2508	251.6290	501.2667	251.1370	4
12	1477.6369	739.3221			1459.6264	730.3168	D	***390.2347***	195.6210	373.2082	187.1077	372.2241	186.6157	3
13	1605.7319	803.3696	1588.7053	794.8563	1587.7213	794.3643	K	275.2078	138.1075	258.1812	129.5942			2
14							K	147.1128	74.0600	130.0863	65.5468			1

MS/MS Fragmentation of **GELLEAIKR** found in **SODM**

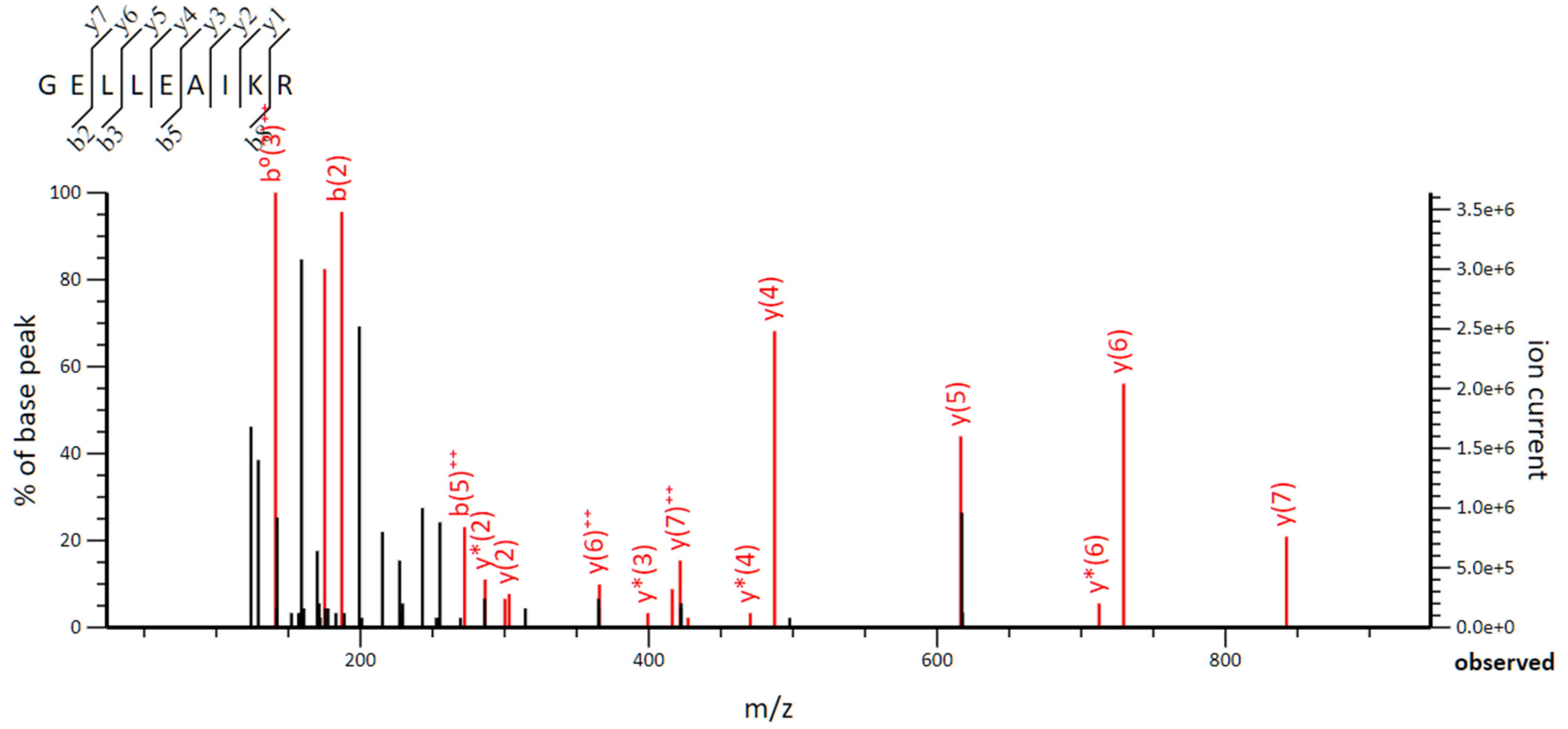

Monoisotopic mass of neutral peptide Mr(calc): 1027.6026
Ions Score: 51 **Expect:** 0.00055
Matches : 20/72 fragment ions using 34 most intense peaks

#	b	b^{++}	b*	b^{*++}	b^{0}	b^{0++}	Seq.	y	y^{++}	y*	y^{*++}	y^{0}	y^{0++}	#
1	58.0287	29.5180					G							9
2	**187.0713**	94.0393			169.0608	85.0340	E	971.5884	486.2978	954.5619	477.7846	953.5778	477.2926	8
3	**300.1554**	150.5813			282.1448	141.5761	L	***842.5458***	**421.7765**	825.5193	413.2633	824.5352	412.7713	7
4	413.2395	207.1234			395.2289	198.1181	L	***729.4617***	**365.2345**	712.4352	356.7212	711.4512	356.2292	6
5	542.2821	**271.6447**			524.2715	262.6394	E	***616.3777***	308.6925	599.3511	300.1792	598.3671	299.6872	5
6	613.3192	307.1632			595.3086	298.1579	A	***487.3351***	244.1712	470.3085	235.6579			4
7	726.4032	363.7053			708.3927	354.7000	I	***416.2980***	208.6526	399.2714	200.1394			3
8	854.4982	**427.7527**	837.4716	419.2395	836.4876	418.7475	K	***303.2139***	152.1106	286.1874	143.5973			2
9							R	***175.1190***	88.0631	158.0924	79.5498			1

MS/MS Fragmentation of **GDVTTQVALQPALK** found in **SODM**

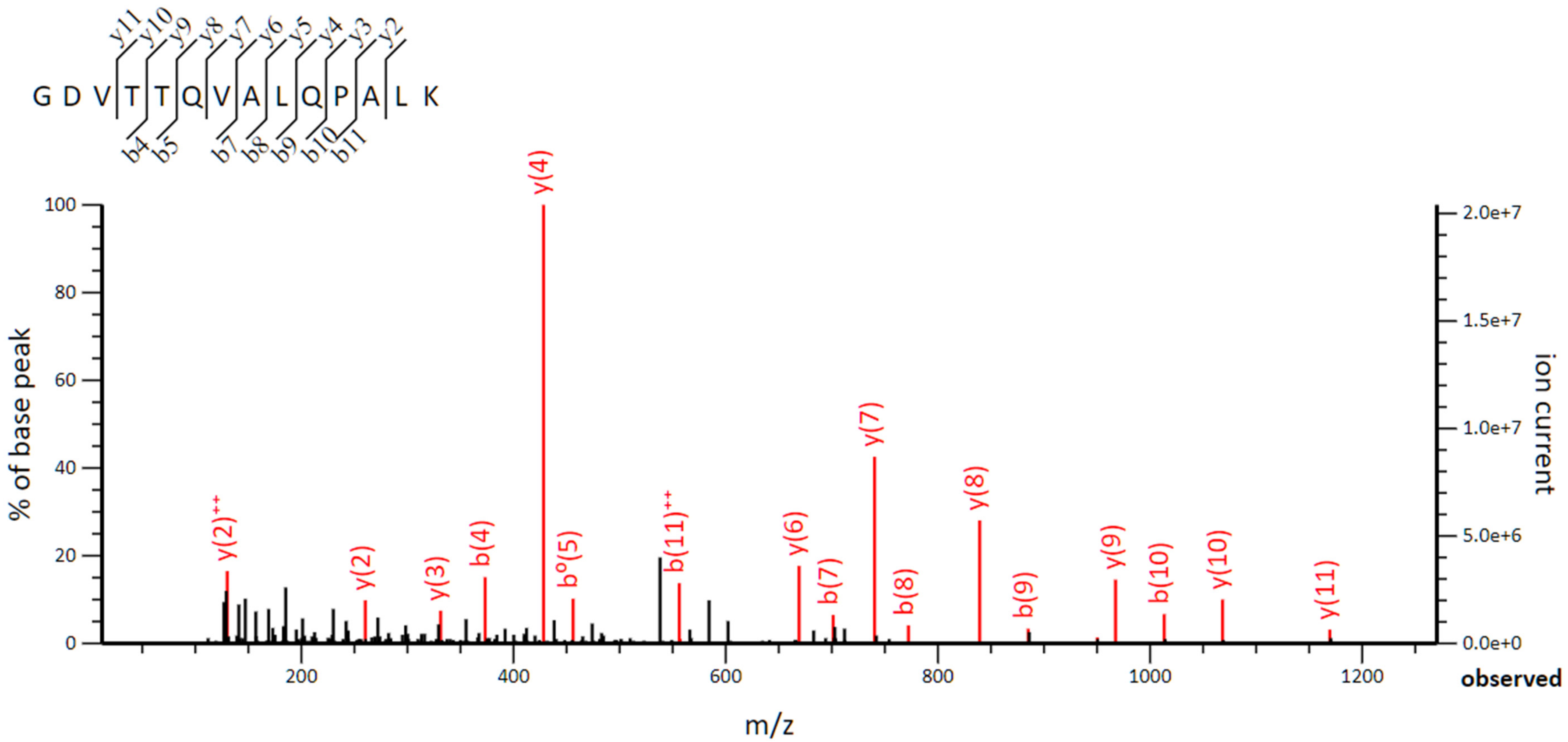

Monoisotopic mass of neutral peptide Mr(calc): 1439.7984
Ions Score: 93 **Expect:** 4.3e-08
Matches : 20/126 fragment ions using 22 most intense peaks

#	b	b^{++}	b*	b*$^{++}$	b^{0}	b^{0++}	Seq.	y	y^{++}	y*	y*$^{++}$	y^{0}	y^{0++}	#
1	58.0287	29.5180					G							14
2	173.0557	87.0315			155.0451	78.0262	D	1383.7842	692.3957	1366.7577	683.8825	1365.7736	683.3905	13
3	272.1241	136.5657			254.1135	127.5604	V	1268.7573	634.8823	1251.7307	626.3690	1250.7467	625.8770	12
4	**373.1718**	187.0895			355.1612	178.0842	T	***1169.6889***	585.3481	1152.6623	576.8348	1151.6783	576.3428	11
5	474.2195	237.6134			456.2089	228.6081	T	***1068.6412***	534.8242	1051.6146	526.3109	1050.6306	525.8189	10
6	602.2780	301.6427	585.2515	293.1294	584.2675	292.6374	Q	***967.5935***	484.3004	950.5669	475.7871			9
7	**701.3464**	351.1769	684.3199	342.6636	683.3359	342.1716	V	***839.5349***	420.2711	822.5084	411.7578			8
8	**772.3836**	386.6954	755.3570	378.1821	754.3730	377.6901	A	***740.4665***	370.7369	723.4400	362.2236			7
9	**885.4676**	443.2375	868.4411	434.7242	867.4571	434.2322	L	***669.4294***	335.2183	652.4028	326.7051			6
10	**1013.5262**	507.2667	996.4997	498.7535	995.5156	498.2615	Q	***556.3453***	278.6763	539.3188	270.1630			5
11	1110.5790	555.7931	1093.5524	547.2798	1092.5684	546.7878	P	***428.2867***	214.6470	411.2602	206.1337			4
12	1181.6161	591.3117	1164.5895	582.7984	1163.6055	582.3064	A	***331.2340***	166.1206	314.2074	157.6074			3
13	1294.7001	647.8537	1277.6736	639.3404	1276.6896	638.8484	L	***260.1969***	130.6021	243.1703	122.0888			2
14							K	147.1128	74.0600	130.0863	65.5468			1

MS/MS Fragmentation of **LAVSQVPR** found in **TKT**

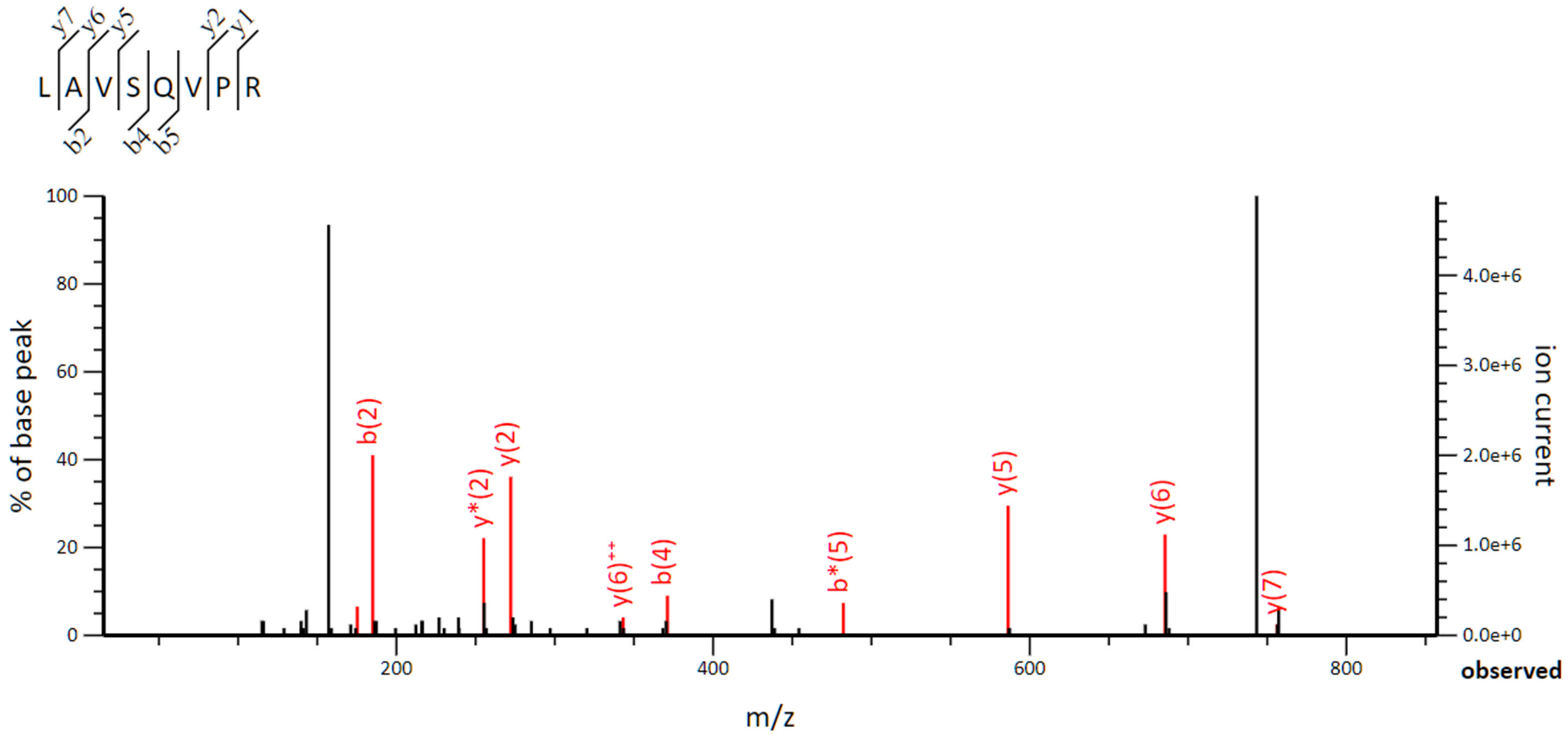

Monoisotopic mass of neutral peptide Mr(calc): 868.5130
Ions Score: 40 **Expect:** 0.0022
Matches : 12/62 fragment ions using 21 most intense peaks

#	b	b^{++}	b*	b^{*++}	b^0	b^{0++}	Seq.	y	y^{++}	y*	y^{*++}	y^0	y^{0++}	#
1	114.0913	57.5493					L							8
2	**185.1285**	93.0679					A	***756.4363***	378.7218	739.4097	370.2085	738.4257	369.7165	7
3	284.1969	142.6021					V	***685.3991***	343.2032	668.3726	334.6899	667.3886	334.1979	6
4	**371.2289**	186.1181			353.2183	177.1128	S	***586.3307***	293.6690	569.3042	285.1557	568.3202	284.6637	5
5	499.2875	250.1474	482.2609	241.6341	481.2769	241.1421	Q	499.2987	250.1530	482.2722	241.6397			4
6	598.3559	299.6816	581.3293	291.1683	580.3453	290.6763	V	***371.2401***	186.1237	354.2136	177.6104			3
7	695.4087	348.2080	678.3821	339.6947	677.3981	339.2027	P	***272.1717***	136.5895	255.1452	128.0762			2
8							R	***175.1190***	88.0631	158.0924	79.5498			1

MS/MS Fragmentation of **HQPTAIIAK** found in **TKT**

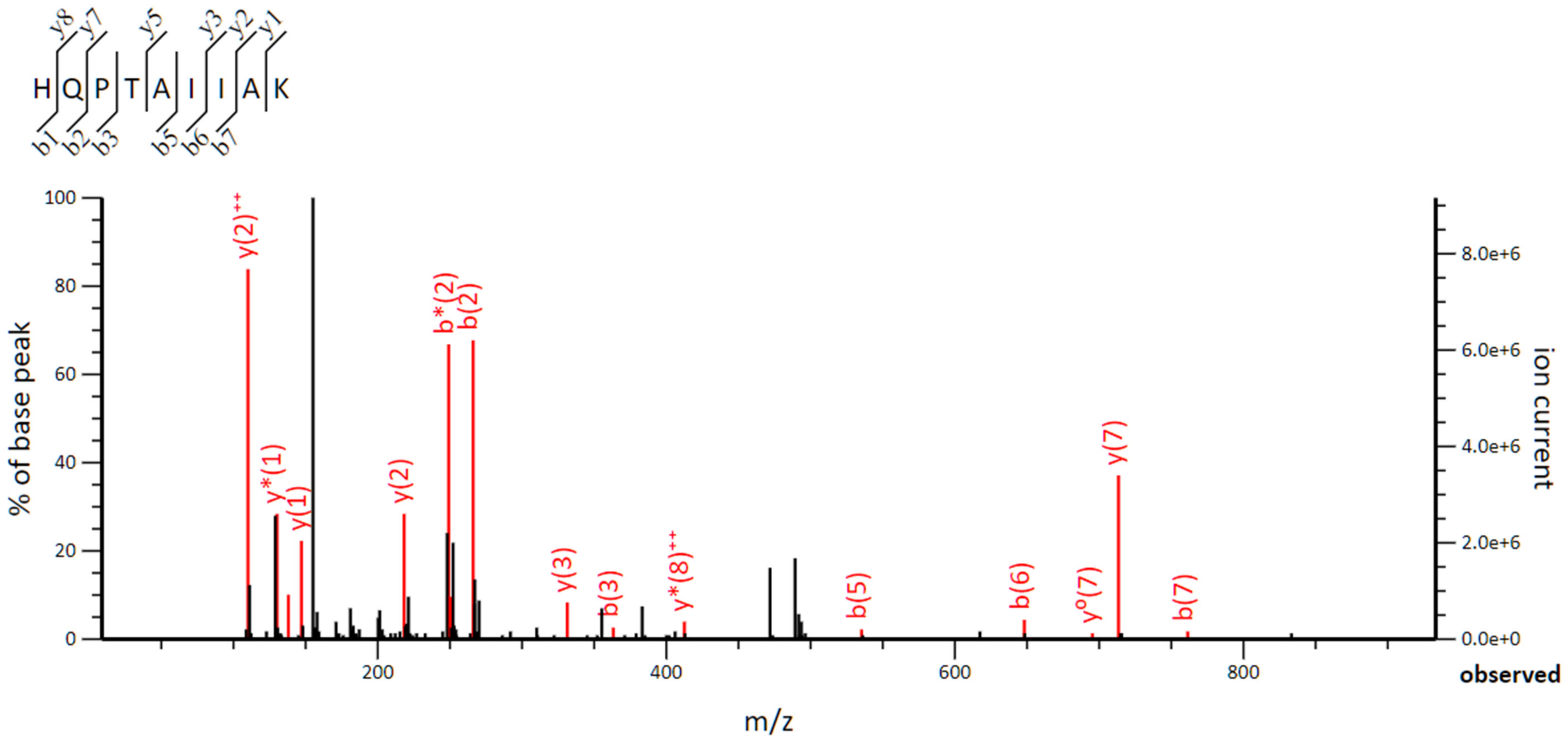

Monoisotopic mass of neutral peptide Mr(calc): 977.5658
Ions Score: 29 **Expect:** 0.028
Matches : 17/78 fragment ions using 40 most intense peaks

#	b	b++	b*	b*++	b0	b0++	Seq.	y	y++	y*	y*++	y0	y0++	#
1	***138.0662***	69.5367					H							9
2	***266.1248***	133.5660	249.0982	125.0527			Q	841.5142	421.2607	824.4876	412.7475	823.5036	412.2554	8
3	***363.1775***	182.0924	346.1510	173.5791			P	**713.4556**	357.2314	696.4291	348.7182	695.4450	348.2262	7
4	464.2252	232.6162	447.1987	224.1030	446.2146	223.6110	T	616.4028	308.7051	599.3763	300.1918	598.3923	299.6998	6
5	***535.2623***	268.1348	518.2358	259.6215	517.2518	259.1295	A	515.3552	258.1812	498.3286	249.6679			5
6	***648.3464***	324.6768	631.3198	316.1636	630.3358	315.6715	I	444.3180	222.6627	427.2915	214.1494			4
7	***761.4305***	381.2189	744.4039	372.7056	743.4199	372.2136	I	**331.2340**	166.1206	314.2074	157.6074			3
8	832.4676	416.7374	815.4410	408.2241	814.4570	407.7321	A	**218.1499**	109.5786	201.1234	101.0653			2
9							K	**147.1128**	74.0600	130.0863	65.5468			1
